# Giant polarization ripple in transverse pyroelectricity

**DOI:** 10.1038/s41467-023-35900-x

**Published:** 2023-01-26

**Authors:** Yi Zhou, Tianpeng Ding, Jun Guo, Guoqiang Xu, Mingqiang Cheng, Chen Zhang, Xiao-Qiao Wang, Wanheng Lu, Wei Li Ong, Jiangyu Li, Jiaqing He, Cheng-Wei Qiu, Ghim Wei Ho

**Affiliations:** 1 Department of Electrical and Computer Engineering, National University of Singapore, Singapore, Singapore; 2 Department of Physics, Southern University of Science and Technology, Shenzhen, China; 3 School of Electronic Science and Engineering, State Key Laboratory of Electronic Thin Film and Integrated Devices, University of Electronic Science and Technology of China, Chengdu, China; 4 Department of Materials Science and Engineering, Southern University of Science and Technology, Shenzhen, China; 5 Department of Materials Science and Engineering, National University of Singapore, Singapore, Singapore; 6 Institute of Materials Research and Engineering, A*STAR (Agency for Science, Technology and Research), Singapore, Singapore

**Keywords:** Electronic properties and materials, Metamaterials

## Abstract

Pyroelectricity originates from spontaneous polarization variation, promising in omnipresent non-static thermodynamic energy harvesting. Particularly, changing spontaneous polarization via out-of-plane uniform heat perturbations has been shown in solar pyroelectrics. However, these approaches present unequivocal inefficiency due to spatially coupled low temperature change and duration along the longitudinal direction. Here we demonstrate unconventional giant polarization ripples in transverse pyroelectrics, without increasing the total energy input, into electricity with an efficiency of 5-fold of conventional longitudinal counterparts. The non-uniform graded temperature variation arises from decoupled heat localization and propagation, leading to anomalous in-plane heat perturbation (29-fold) and enhanced thermal disequilibrium effects. This in turn triggers an augmented polarization ripple, fundamentally enabling unprecedented electricity generation performance. Notably, the device generates a power density of 38 mW m^−2^ at 1 sun illumination, which is competitive with solar thermoelectrics and ferrophotovoltaics. Our findings provide a viable paradigm, not only for universal practical pyroelectric heat harvesting but for flexible manipulation of transverse heat transfer towards sustainable energy harvesting and management.

## Abstract (web summary)

Pyroelectricity promises viable heat harvesting and sensing. Here, the authors identify transverse polarization ripple in pyroelectrics via heat localization and propagation decoupling and offer competitive power output to solar thermoelectrics.

## Introduction

The pyroelectric effect which generates electricity via spontaneous polarization change under temporal temperature fluctuations, is a promising approach to scavenge the underexplored diffused heat of unstable, non-static thermodynamic processes. The pyroelectricity is typically given by $p=\partial P/\partial T$ and $P={P}_{{{{{{\rm{S}}}}}}}+{P}_{{{{{{\rm{field}}}}}}}$ from the electric dipole moment change per unit volume of polar materials^1–3^. Here $p$ is the pyroelectric coefficient, ${P}_{{{{{{\rm{S}}}}}}}$ and ${P}_{{{{{{\rm{field}}}}}}}$ are intrinsic spontaneous polarization (mainly contributed by the primary pyroelectricity) and electrical polarization with respect to applied thermal and electric fields, respectively. Though the $p$ of pyroelectric materials has almost been pushed to the upper limit^4,5^, it is still a long way from being practically deployed for waste/solar heat harvesting on account of the low energy conversion efficiency.

Recently, electric-field-free pyroelectrics (${P}_{{{{{{\rm{field}}}}}}}=0$) have presented an increase in generated power density from 10 to 10^3^ μW m^−2^ for low-grade solar heat harvesting via out-of-plane heat variation^6–8^. However, these conventional (CONV) devices harness solar heat homogenously, limited by simultaneous heat conversion and transfer/dissipation along the longitudinal direction^8–10^. This spatially coupled thermodynamic process results in small heat gradients (Δ*T*), non-static temperature changes (d*T*/d*t*), and thermal disequilibrium durations (d*t*). Thereby not only the temperature-dependent ${P}_{{{{{{\rm{S}}}}}}}$ (i.e., surface charge density from dipole moment change) was restricted, but also the pyroelectric voltage (*V*(*t*) ∝ *p* and Δ*T*) and current (*I*(*t*) ∝ *p* and d*T*/d*t*)^11^. Consequently, traditional pyroelectrics are unfeasible for intermittent low-grade solar heat harvest, aiming to achieve high electricity output based on inconsiderable or unstructured ambient energy input. Although many efforts have been devoted to boosting pyroelectricity, e.g., polarization change via applied electric field (${P}_{{{{{{\rm{field}}}}}}}\ne 0$)^12–14^, modulating thermodynamic cycles^15,16^, tailoring material properties^5,17,18^, or hybridization with other effects/mechanisms^19–21^, the harvesting of the prevalent non-static heat is making little headway due to the additional energy penalty or device complexity and instability. Fortunately, controllable transverse heat localization and/or redistribution has been proven promising in photothermal interfacial heating and nonlinear light-modulated solar vaporization;^22–25^ there are a few, if any, studies on efficient pyroelectricity via heat transfer manipulation.

In this work, we report the exploitation of polarization ripple arising from in-plane temperature fluctuations of transverse pyroelectricity generator (TPG) to overcome the current impediment for practical thermodynamic energy conversion. Distinct from the typical longitudinal uniform photothermal pattern, the decoupling of modulated heat localization and thermodynamic disequilibrium leads to anomalous in-plane diffusive heat conduction towards non-uniform graded temperature fields. Accordingly, this engenders a giant transverse d*T*/d*t* and polarization ripple. Without consuming additional solar/electrical energies, altering pyroelectric coefficients, or tailoring materials properties, the TPG system can convert the otherwise unavailing heat into usable electricity with significantly improved efficiency of one order of magnitude higher than conventional devices. As a result, the output current and voltage of a single TPG are upgraded respectively by ~90% and ~185%, facilitating an achieved power density of 38 mW m^−2^, the record high at a low illumination of 10 mW cm^−2^, even after 1000 heating/cooling repetitive cycles. Our work paves a feasible way to advance the practical implementation of pyroelectric systems for non-static or quasi-static sustainable heat harvesting, and it is an inspiration for manipulating transverse thermoelectricity and thermal metasurfaces.

## Results

**Fig. 1:**
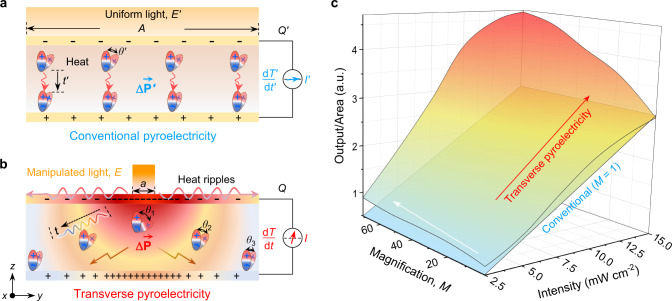
The conceptual mechanistic of TPG in comparison with a conventional device. **a** Schematic depiction of conventional (CONV) pyroelectric device. **b** Schematic illustration of TPG. *M* = *A*/*a* stands for the magnification of low-grade solar heat localization for TPG surface area (*A*) compared with irradiation area (*a*), where *E* = *ME’, I* > *I’*, Δ*P* > Δ*P’, θ*_1_ > *θ’ > θ*_3_, *Q* > *Q’*, d*T*/d*t* > d*T’*/d*t’* and *t* > *t’*. The polarization follows the direction of temporal temperature variations. **c** Normalized output for conventional (back, *M* = 1) and TPG devices under different irradiation intensities and magnifications.

The conceptual mechanistic of pyroelectrics under uniform solar heating is schematically presented in Fig. 1. The heat flux and fluctuation of a conventional device (Fig. 1a) typically propagate and dissipate in the out-of-plane direction, i.e., the *z*-direction along with the device thickness, and the irradiation area equals the device area (*A*), stemming a constant magnification (denoted as the conventional scenario, *M* = 1). Contrarily, the proposed transverse pyroelectric generator (Fig. 1b) harvests the solar heat in a confined area (TPG, *M* > 1), inducing atypical non-uniform heat delivery from the hotspot to the non-irradiation area. This spatially decoupled heat localization and propagation engender fluctuated heat variation and polarization ripple along the transverse direction (i.e., *x–y* plane) to the non-irradiated peripheral part (Fig. 1b), which also prolongs the temperature variation time in the pyroelectric system. Moreover, according to pyroelectric fundamentals^26^, the local intense heat variation (d*T*/d*t*) creates a giant polarization ripple (i.e., Δ*P*_S_ or larger dipole moment shift, *θ*), attributed to higher electrostatic induced surface charge density (*σ*). Despite having the irradiation area reduced, our TPG outperforms the conventional device and contributes to an unprecedented output enhancement (Fig. 1c) under identical illuminations and magnifications. This is ascribed to the augmented non-uniform transverse polarization ripple and prolonged heat propagation duration. In addition, the cooling process shares a similar thermal propagating phenomenon with the heating process while there is no consistent heat input to the system, which, likewise, results in higher pyroelectricity than the traditional counterpart. The unconventional transverse heat confinement provides the basis of triggering travelling thermal packages valid for heating/cooling processes which hold enormous potential for advancing energy efficiency and autonomy in system operation.

### Identification of transverse polarization ripple

**Fig. 2:**
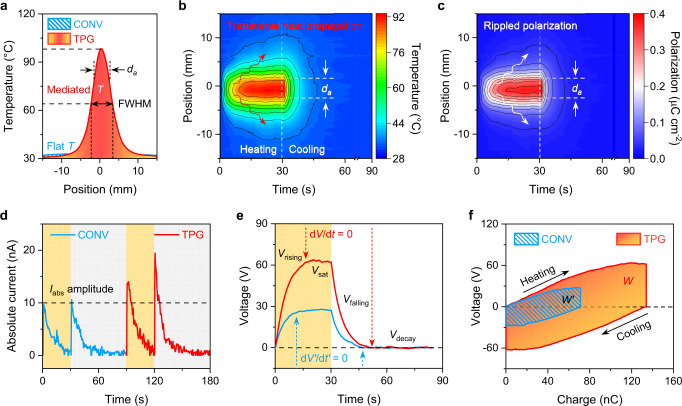
Identification of temperature-driven polarization ripple in transverse pyroelectricity. **a** Temperature profiles of a single conventional (stripe) and TPG devices. The upper and lower dashed lines refer to the maximum and half maximum temperatures of the TPG device. FWHM stands for the full width at half maximum of the temperature distribution. **b**, **c** Measured spatiotemporal temperatures (**b**) and polarization ripples (**c**) of TPG device. **d** The absolute current response for conventional (blue lines) and TPG (red lines) devices. The dashed line marks the maximum amplitude of absolute current for the conventional device. **e** The voltage response of a single conventional and TPG device. The time-differential voltage indicates the temperature-dependent voltage dynamics under the rising, saturation, falling, and decay stages. **f** The voltage varies with charge, and harvested energy (integration as shown in the shadow area) of a single conventional (stripe) and TPG devices, where *W* > *W’* indicates the harvested energy of TPG is greater than that of conventional device. The dashed line masks zero voltage at thermal equilibrium, and the equivalent voltage relative to the cooling stage is negative, specifying the voltage consumption/decrease shown in Fig. 2e and Supplementary Fig. 7. The illumination intensity for measurement is 0.1 sun.

To elucidate how the macroscopic, in-plane, solar heat manipulation impacts the temporal temperature perturbation and transverse polarization change, i.e., solar heat localization-propagation decoupling, as well as underlying pyroelectric characteristics, a thermal and electrical measurement setup was constructed to evaluate the device output (Supplementary Figs. 1 and 2a). Then, a polyvinylidene difluoride (PVDF) thin film coated with solar absorber materials was employed as the device for the measurement (Supplementary Fig. 2b, see Methods for device fabrication and characterization). Remarkably, the temperature profiles of the TPG reveal a temperature increment of 206.3% and heat variation (d*T*/d*t*) by two orders of magnitude (29 folds) higher at the irradiated local hotspot, in comparison to the uniform heat distribution of the conventional device (Fig. 2a, Supplementary Fig. 2c). Moreover, the temperature FWHM (full width at half maximum) of TPG is comparable to the diameter of irradiated area (*d*_a_), validating the phenomenon of solar-to-heat confinement, and well-manipulated in-plane heat ripple fluctuation across the TPG. In terms of the spatiotemporal temperature distribution of TPG under heating/cooling processes (Fig. 2b), the multiple temperature contours affirm the delineated thermal ripple fluctuation of solar heat from the illuminated hotspot to circumjacent areas. It is also apparent that the heat dissipates far more sharply for cooling relative to the heating process. The observed inhomogeneous heat transfer differentiates from the typical out-of-plane nearly constant heat flux under uniform solar heating (Supplementary Fig. 3a, b). Furthermore, the pyroelectric coefficient (Supplementary Fig. 3c, Supplementary Note 1) of a single PVDF-based device was measured to quantify the extent of polarization or surface charge density attainable from the in-plane heat variation. In specific, the PVDF-based device with a parallel-plate capacitor configuration (Supplementary Fig. 4) was used to extract the pyroelectric coefficient under varied light illuminations, and the polarization anisotropy was not considered in this measurement (detailed discussion see Supplementary Note 2). We found that the spatial and temporal polarization ripples (Fig. 2c) conform to the transverse heat variation (Fig. 2a, b) and present a non-uniform distribution around one order of magnitude higher than the conventional polarization in the irradiated area (Supplementary Fig. 3d). Consequently, the overall enlarged heat variation and polarization ripple of TPG not only counteract the output decrease in the non-illuminated area, but also manifest the enhancement in the electric current, voltage, as well as harvested energy (Fig. 2d, f). These outputs are mainly ascribed to the primary pyroelectricity^1,26,27^, as verified in Supplementary Fig. 5 and Supplementary Note 3. Notably, the absolute current (Fig. 2d) of the TPG is boosted by 44.3 % and 83.8% at the heating and cooling stages, respectively, in agreement with the sharper temperature drop during the cooling process in Fig. 2b. The time-dependent current and voltage characteristics (Fig. 2e, Supplementary Figs. 6 and 7) show distinctive electric rising (*V*_rising_), saturation (*V*_sat_), falling (*V*_falling_), decay (*V*_decay_) behaviours of conventional and TPG devices. With respect to thermodynamics and pyroelectric mechanistic (Fig. 1a and b, Supplementary Fig. 2a), the voltage and current responses can be quantified by the thermal relaxation time (*τ*)^26^, i.e., *τ* = *l*^2^*C*_T_/*κ*, where *l* is the length in the direction of heat conduction, *C*_T_ and *κ* refer to heat capacitance in volume and thermal conductivity of pyroelectric materials. Consequently, the transverse heat perturbation with a larger heat conduction length protracts the thermal relaxation time for augmented energy scavenging, contributing to a longer pyroelectric working span. The enhanced TPG output is also quantified using voltage-charge integration (Fig. 2f), where the work done (*W*) or energy harvested can be estimated by the electrical potential (*V*) and transferred charges (*Q*) in a capacitor^28^. Under fixed solar heating/cooling processes, the *V*-*Q* characteristics of the TPG unit demonstrate considerably higher voltage (79.4 V) and charge accumulations (0.13 μC), as well as collected energy (10.6 μJ) than the conventional device.

### Finite element modelling and experimental verification

**Fig. 3:**
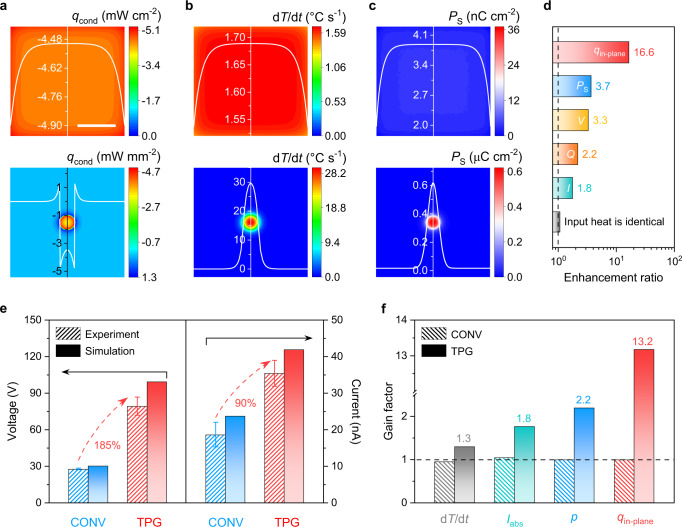
Finite element analysis and experimental verification. **a–c** Simulation profiles of in-plane conductive heat flux (**a**), temporal temperature variation (**b**), and polarization (**c**) for conventional (upper rows) and TPG (bottom rows) devices. Scale bar: 10 mm. **d** Enhancement ratio of TPG/conventional devices estimated from simulation profiles. The dashed line specifies 1.0 for the ratio of TPG/conventional devices. **e** Output voltage (the left two columns) and current (the right two columns) of conventional and TPG devices. The experimental and simulated incident illumination intensities are fixed at 0.1 sun. **f** Gain factor of conventional and TPG devices under cooling/heating measurements. The dashed line marks 1.0 for a typical device at consistent heating/cooling processes.

To explicate the underlying mechanism of unconventional polarization ripple in transverse pyroelectricity, the electrical output of TPG under solar heating/cooling cycles from numerical modelling and experiments was investigated and compared. First, the thermodynamics and electrostatics of conventional and TPG devices under an illumination of 10 mW cm^−2^ were analyzed using COMSOL Multiphysics software (Supplementary Fig. 8, Supplementary Table 1, see Methods for finite element modelling). The conventional device driven by the out-of-plane temperature variations suggests a uniform heat transfer diagram (upper rows, Fig. 3a, b, and Supplementary Fig. 9a–e) and electrical output (upper rows, Fig. 3c, Supplementary Fig. 10a, b) owing to comparably balanced illumination and surrounding heat dissipation^7^. In contrast, as shown in the bottom rows, the quasi-static conductive heat flux (${q}_{{{{{{\rm{cond}}}}}}}$) driven by the local solar heat input (${q}_{{{{{{\rm{input}}}}}}}$), as well as ambient convective (${q}_{{{{{{\rm{conv}}}}}}}$) and radiative (${q}_{{{{{{\rm{rad}}}}}}}$) heat fluxes (Fig. 3a, Supplementary Fig. 9a–e) bring forth unexpected in-plane heat perturbation (d*T*/d*t*) and rippled transverse polarization (*P*_S_) (Fig. 3b, c), towards current density and charge density multiplications (Supplementary Fig. 10c, d). Fundamentally, the decoupled heat confinement and dissipation of TPG governed by the anomalous in-plane heat conduction (i.e., ${q}_{{{{{{\rm{in}}}}}}-{{{{{\rm{plane}}}}}}}$), non-uniform heat convection and radiation (Supplementary Fig. 9e,f) have resulted in self-propagating travelling thermal packages that promote the dipole moment changes^29–31^. Thereby the increment of electrostatic induced charge in the irradiated area (*a*) surpasses the reduction in the non-irradiated area (*A* - *a*). As confirmed by the enhancement ratio of TPG/conventional from the simulation profiles (Fig. 3d, Supplementary Figs. 9f and 10e), the output of TPG was improved by one to two orders of magnitude compared with conventional devices under an identical solar heat input. These results are consistent with the measured data (Figs. 2b–f and 3e) and well-supported by the underlying fundamental analysis from macroscopic heat transfer and lattice dynamics of pyroelectricity (see Methods for detailed discussion). In specific, the measured voltage and current of a single TPG were enlarged by 185% and 90% (Fig. 3e), respectively. The current, voltage, and charge vary synchronously, which is commensurate with the non-static temperature and uncover exceedingly large response and amplitude (Fig. 3d, e, and Supplementary Fig. 11). Furthermore, to determine the extent and contribution of TPG output enhancement under solar heating and cooling separately, we introduced a descriptor, i.e., gain factor, to normalize and quantify the heat variation and electrical output of the cooling relative to the heating process (Supplementary Table 2). Explicitly, the gain factor is around 1.0 under conventional longitudinal heating/cooling processes;^27,32^ however, a modulated cooling process in our TPG (e.g., non-uniform spatiotemporal temperature distribution, anomalous heat conduction.) can substantially facilitate the gain factor >1.0, implying enlarged heat perturbation and dissipation towards output gain enhancement in the cooling process. Different from the monotonic gain factor of conventional device (around 1.0; Fig. 3f, Supplementary Table 2), the values of d*T*/d*t* and *p* enlarged by unconventional in-plane quasi-static conductive heat flux (${q}_{{{{{{\rm{in}}}}}}-{{{{{\rm{plane}}}}}}}$) raises the current of TPG (Figs. 2d and 3f). Hence, the thermodynamic behaviour in the cooling phase contributes to the electrical output of TPG is considerably larger than that of the heating process (e.g., ${q}_{{{{{{\rm{in}}}}}}-{{{{{\rm{plane}}}}}}}$ was boosted by ~13 folds). These findings draw an unconventional insight into the understanding of synergistic modulation of pyroelectric thermodynamics, electrostatics, and corresponding heat harvesting performance.

### Enhancement of polarization ripple in transverse pyroelectricity

**Fig. 4:**
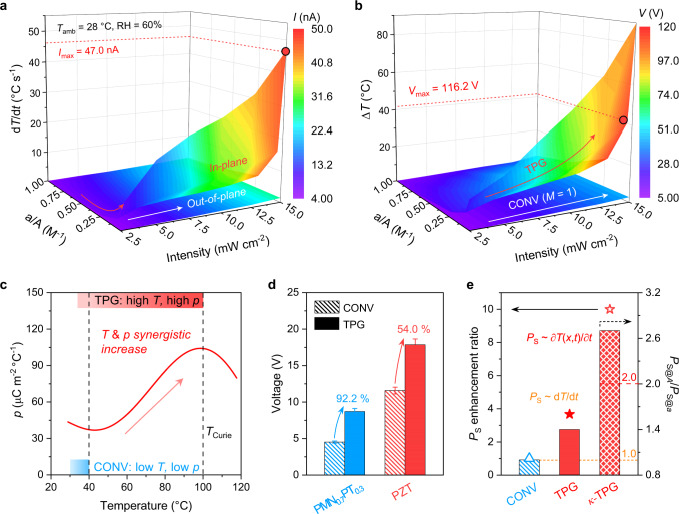
Enhancement of rippled polarization and TPG output. **a**, **b** Pyroelectric current (**a**) and voltage (**b**) of single conventional (*M* = 1) and TPG devices at various irradiation areas and solar intensities. **c** Temperature and pyroelectric coefficient synergistic manipulations from conventional to transverse (TPG) pyroelectrics for typical low-*T*_Curie_ (<100 °C) devices under heating/cooling variations. The coloured blocks indicate the operating temperature range for conventional and TPG devices at identical solar illuminations, respectively. **d** Current and voltage enlargement of single inorganic conventional and TPG devices at 0.1 sun. PMN_0.7_PT_0.3_, 0.7Pb(Mg_1/3_Nb_2/3_)O_3_−0.3PbTiO_3_; PZT, Pb(Ti,Zr)O_3_. **e** Polarization enhancement ratio versus conventional design (left, *P*_S_/*P*_S@CONV_) and polarization in the device area and illuminated area (right, *P*_S@A_/*P*_S@a_).

We further optimized the current and voltage amplification of TPG at different illuminations and magnifications to extract the maximum electrical output. The magnification (*M*) was modulated by varying the back image distance (BID) from 0 to 48 mm with an interval of 8 mm. The irradiation area (*a*) was recorded from the light spot, *M* was calculated from the device area (*A*) and *a* (Supplementary Fig. 12). The measured current and voltage under tunable BID values were plotted in Supplementary Fig. 13a and 13b, respectively. It is observed that both the current and voltage reach the peak at BID = 32 mm under different intensities, i.e., the focus position is optimum for manipulated in-plane disequilibrium heat perturbation (d*T*/d*t*) towards boosted transverse polarization ripples. On the other hand, the intensity-dependent current and voltage vary linearly for both conventional and TPG devices (Supplementary Fig. 13c and 13d), in agreement with the pyroelectric current and voltage formulae^11^. However, the thermo-current density (36.2 nA cm^−2^ sun^−1^) and thermo-voltage density (86.1 V cm^−2^ sun^−1^) of the TPG are two folds higher than that of the conventional device, indicating a more efficient thermo-electrical output under identical input. The current and voltage enhancement, as well as the internal resistance, are consistent across varied solar intensities (Supplementary Fig. 14). The optimized current and voltage derived from the various magnifications and intensities were plotted in Fig. 4a, b. Evidently, the enhanced electrical output of conventional device is limited and varies nominally across different magnifications and intensities. In contrast, a maximum current of 47 nA (density of 5.2 nA cm^−2^) and voltage of 116.2 V (density of 13 V cm^−2^) for the TPG are achieved at a maximized magnification of 71.7 under ~ 15 mW cm^−2^ illumination. These results corroborate that the proposed TPG yields amplified responses and is promising for upcycling solar heat when operating at ultra-low-grade illumination intensities (1-10 mW cm^−2^). Also, the TPG output versus scaling down PVDF dimensions reveals the enhanced transverse pyroelectricity is less size-dependent and slightly fluctuates with the magnification, especially at an optimum configuration (i.e., BID = 32 mm and *d*_a_ = 4 mm, Supplementary Fig. 15). Noticeably, according to the nature of nonlinear temperature dependence of $p$^11,33,34^, the synergistic manipulation of harvested solar heat and *P*_S_ from conventional to transverse pyroelectricity (Fig. 4c) is universal for general polar materials below the Curie temperature (*T*_Curie_). In particular, uplifting the varying temperature in a thermodynamic process to a higher $p$ range (i.e., close to but less than the *T*_Curie_) benefits upgrading the energy quality and improves the energy harvesting efficiency^35^. In this regard, we demonstrated the transverse pyroelectricity using inorganic PZT (Pb(Ti,Zr)O_3_) and PMN-PT (0.7Pb(Mg_1/3_Nb_2/3_)O_3_−0.3PbTiO_3_) thin films. The boosted current and voltage are ascribed to the in-plane large heat perturbation and rippled polarization (Fig. 4d, Supplementary Fig. 16). Moreover, the enhanced polarization of transverse pyroelectricity versus conventional one (*P*_S_/*P*_S@CONV_) was estimated using finite element methods (Fig. 4e, Supplementary Fig. 17). The dramatical increment of 267% was achieved, which can be further improved to ~ 900% by solely employing asymmetrical configurations with inhomogeneous thermal conductivities (*κ-*TPG) under identical inputs. Meanwhile, the *P*_S_ of non-irradiated area outperforms that of the illuminated hotspot (*P*_S@A_/*P*_S@a_ > 2) for *κ-*TPG with asymmetrical solar absorbers, in contrast to TPG (1<*P*_S@A_/*P*_S@a_ < 2) and conventional devices (*P*_S@A_/*P*_S@a_ = 1). These results suggested that the transverse *P*_S_ determined by the temporal temperature disequilibrium could be further leveraged by manipulating nonlocal heat propagation (where the temperature *T*(*x*, *t*) is of spatiotemporal dependence^36^) and promoting dipole moment change from electron-phonon renormalization^2^.

### Proof-of-concept scalable TPG systems

**Fig. 5:**
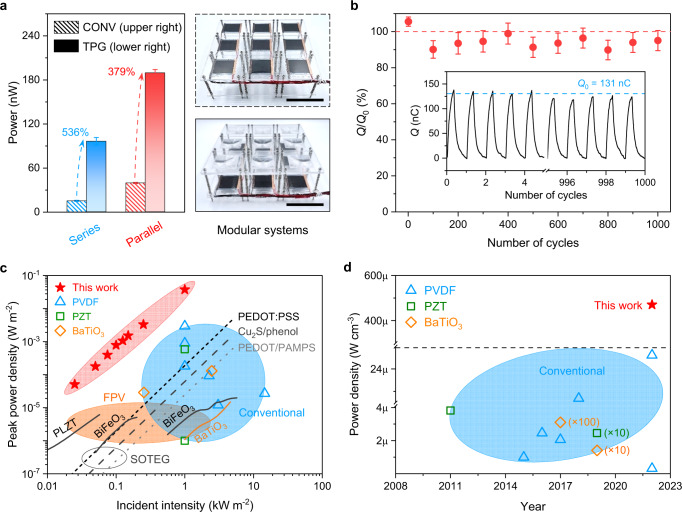
Scalable TPG systems and comparison of power output with conventional devices. **a** The output power for modular conventional (upper right) and TPG (lower right) systems, in series and parallel mode under 0.1 sun. Scale bar: 5 cm. **b** Harvested charge measurement of TPG under 1000 heating/cooling cycles. Inset, the first and last five cycles. *Q*_0_ stands for the initial measured peak charge, as shown in Supplementary Fig. 11f. **c** Comparison of peak power density for proposed TPG (solid scatters) and conventional devices at different solar illuminations. The dashed and dotted lines represent solar organic thermoelectric generation (SOTEG), and the solid lines refer to ferrophotovoltaics (FPV, orange region). **d** Historical view of volumetric power density for conventional devices and TPG results. Blue region: conventional devices. Details of power density comparison in (**c**) and (**d**) can be found in Supplementary Table 3.

To demonstrate the scalability, stability, and practicability of reformative thermal ripple strategy for low-grade solar heat harvesting, we delivered a TPG system consisting of 9 units (Fig. 5a, Supplementary Fig. 18) and evaluated the output power in series and parallel configurations under various load impedances (Supplementary Fig. 19, Supplementary Note 4). The output power increase of 536% and 379% for TPG systems were obtained in series and parallel connections, respectively. The difference in power enhancements between the two modes is mainly attributed to the distinct internal resistance and load impedance (Supplementary Fig. 19)^28^. Besides, the steady output of harvested charges under 1000 heating/cooling cycles (Fig. 5b) attests the negligible leakage current and cycle-independent electrostatics of TPG dissimilar with traditional field-driven pyroelectrics^13,16,26^, thus ensuring continuously high output for stable and sustainable heat harvesting. The state-of-the-art comparison of peak power density, current density, and maximum Carnot efficiency of solar pyroelectrics indicate that the TPG is superior for solar heat harvesting with a peak power density of 1.5 mW m^−2^ (up to 38 mW m^−2^ at 1 sun manipulated by multiple heat ripples, Supplementary Figs. 20 and 21) and maximum Carnot efficiency of 23% (Supplementary Fig. 22) at low intensities, in contrast to conventional devices (Supplementary Note 5, Supplementary Table 3). Moreover, the TPG (Fig. 5c) achieves equivalent power density at low-grade solar illumination as the conventional pyroelectrics (blue region), thermoelectrics, and ferrophotovoltaics (FPV, orange region) that operate at high irradiance. The generated power of TPG in the area and weight metrics is even greater than that of solar organic thermoelectric generators (SOTEG, Supplementary Fig. 23) and has recorded the highest volumetric power density up to now (Fig. 5d). These achievements offer a feasible solution for practicable solar heat harvesting by upgrading ubiquitously attenuated or distributed illumination towards in-plane decoupled heat localization and propagation. Unlike conventional solar pyroelectric^37^ and thermoelectric^38^ mechanistic, where the energy efficiency is proportional to the incident illumination that dissipates simultaneously and uniformly along the longitudinal direction with required sample holders or heat sinks, resulting in non-pragmatic low power generation. We also performed a shadowed outdoor test (under a tree) that was subjected to sunlight fluctuations. The solar flux, wind speed, and parallel current, as well as ambient parameters, were recorded concurrently (Supplementary Fig. 24). The electrical output is induced by the natural sunlight-focused hotspot at TPG systems and wind-driven heat convection (Supplementary Movie 1). Notably, the measured maximum current density is around 36.2 μA m^−2^ (Supplementary Fig. 24c), and estimated voltage density is ~ 36.2 kV m^−2^ (obtained from the linear ohmic characteristics at various intensities shown in Supplementary Fig. 14 and internal resistance in Supplementary Fig. 19b) at ~ 0.1 sun. Such low solar intensity is common but overlooked day-to-day illumination in non-equator countries/areas. These results agree with the superposition of single TPG under lab measurement, signifying easily scalable and optically configured low-grade solar heat harvesting.

## Discussion

In conclusion, we investigated a non-uniform polarization ripple in transverse pyroelectricity to address the long-standing challenge in low-grade unstructured or non-static heat harvesting. The PVDF-based TPG with heat ripples achieves the highest power density record of 38 mW m^−2^ at 1 sun thus far, significantly outperforming conventional approaches with >5 folds higher efficiency. The decoupled solar-to-heat confinement and anomalous in-plane temperature oscillations account for such remarkable enhancement effects in thermo-electricity conversion. This synergistically intensifies the temporal temperature fluctuation induced spatial polarization ripple and prolongs the instantaneous heat propagation span relevant to heat disequilibrium. Further development in transverse diffusive heat transfer manipulation, including asymmetrical solar absorbers, thermal metasurfaces, and graded thermal conductors, is promising to potentially advance the polarization ripple for efficient heat-to-electricity harvesting. Our low-grade heat upcycling through controlled heat redistribution also opens pathways to other energy harvesting/management frontiers, e.g., transverse thermoelectricity, nonlinear heat manipulation, and thin-film heat recovery/management.

## Methods

### TPG fabrication and characterization

A ferroelectric PVDF (Fils Co., Ltd.) thin film with a dimension of 30 mm × 30 mm × 80 μm was used for solar heat harvesting. The top and bottom sides of PVDF thin film were deposited with poly(3,4-ethylenedioxythiophene) (PEDOT) to form a typical sandwich-structured parallel plate capacitor (i.e., PEDOT/PVDF/PEDOT). Two copper tapes (width, 2 mm; thickness, 50 μm) were affixed to the bottom and top surfaces of PVDF separately for electrically conductive wires. To enhance the solar-to-heat absorption, carbon nanotubes (CNT) solar absorber was coated on the top side of PVDF device using the evaporation coating method, and details can be found in the previous work^8^. Besides, inorganic ferroelectric thin films PMN_0.7_PT_0.3_ (KJMTI) were deposited with Au electrodes on the upper and bottom surfaces. The CNT solar absorber was prepared, and electrically conductive wires were attached to the inorganic thin films PMN_0.7_PT_0.3_ and PZT (Daobo Ultrasonics), following the as-mentioned methods. After that, a single device was mounted onto a squared-matched hollow acrylic cantilever for measurement. Moreover, the absorbance and transmittance spectra of PVDF, PVDF/CNT, and acrylic Fresnel lens were obtained using a UV-VIS-NIR spectrophotometer (UV-3600, Shimadzu). The morphology of the Fresnel lens was characterized using a microscope camera (HY3307B, HAYEAR).

### Thermal and electrical measurement of TPG

The thermal and electrical measurement of TPG were conducted simultaneously in a homemade semi-closed system (Supplementary Fig. 1), where a Xenon lamp (CEL-PE300E-3A, CEAULIGHT), an infrared camera (E50, FLIR Systems), as well as an electrometer (6514, Keithley) were positioned in a semi-closed moisture-proof cabinet (controlled temperature and relative humidity). The Xenon lamp was utilized as a solar simulator with an illumination intensity of ~ 2.5 to 15.0 mW cm^−2^ (maximum value of 125.0 mW cm^−2^) under room temperature (~ 28 °C) and relativity humidity (~ 60%). The solar irradiation intensity was measured by a solar power meter (1333 R, TES). An electronic shutter (GCI-73, Daheng Optics) and Fresnel lens were introduced into the light trace for modulating the heating/cooling time duration and incident intensity magnification (*M*). The heating/cooling time duration ratio was fixed at 0.5 (light on, 30 s; light off, 60 s) for the whole test. The back image distance (lens-device distance) is tunable for intensity magnification manipulation. The whole area of Fresnel lens and device remains consistent. The electrometer, coupled with LabVIEW software, was used to record the electrical output variation continuously over time. The maximum input/measurable voltage of the electrometer was ±210 V. Beyond the measurable voltage range, the device was connected to a matched resistor, and the voltage was indirectly calculated by the measured current. Besides, the FLIR camera below the pyroelectric device and FLIR Tools+ software were used to capture the infrared image and surface temperature. The temperature data used in this work is the average value processed from the illumination area of the device. Each measurement was performed under 10 cycles of temporal heating/cooling controlled by the electronic shutter, and the error bar was calculated from the standard deviation of peak-to-peak outputs. For the stability and durability test of the developed TPG, the number of cycles was 1000, and the light on/off ratio was 0.5. Here the light on/off time duration and the number of cycles was controlled by an electronic shutter, and other conditions remained consistent with normal device measurement.

### Finite element analysis of TPG

The thermal and electrical characteristics of PVDF-based conventional and TPG devices were performed by the finite element method using COMSOL Multiphysics coupled with Heat Transfer and Electrostatics modules. The measured device dimension and other parameters selected from the COMSOL materials library were utilized in the simulation, as indicated in Supplemental Table 1. The pyroelectric coefficient *p*(*T*) = 1.2239E-7*T* ^5^ − 4.68522E-5*T* ^4^ + 6.16E-3*T* ^3^ − 0.32908 *T* ^2^ + 6.91557 *T*, fitted function from as-measured results was used for the analysis. To reveal the heat ripple propagation and d*T*/d*t*-induced pyroelectric electrostatics, the solar-heat generation process, i.e., light absorption of device, was simplified into a solid heat transfer model. The input thermal energy from the solar incident irradiation was approximated to a heat penetration depth with a consideration of PVDF/CNT absorbance, defined as the boundary heat source (${q}_{{{{{{\rm{input}}}}}}}$ is 10 mW cm^−2^), so the governed equations for instantaneous heat transfer, heat conduction, and heat dissipation are given from a view of the first law of thermodynamics

1 $$\rho {C}_{{{{{{\rm{p}}}}}}}\frac{\partial T}{\partial t}+\rho {C}_{{{{{{\rm{p}}}}}}}{{{{{\boldsymbol{u}}}}}} \cdot \nabla T+\nabla \cdot {{{{{{\boldsymbol{q}}}}}}}_{{{{{{\bf{cond}}}}}}}=Q,$$

2 $${{{{{{\boldsymbol{q}}}}}}}_{{{{{{\bf{cond}}}}}}}=-\kappa \nabla T,$$

where *ρ* and *C*_*p*_ stand for the density and constant-pressure heat capacity, respectively. ${{{{{{\boldsymbol{q}}}}}}}_{{{{{{\bf{cond}}}}}}}$ is the quasi-static conductive heat flux, and *κ* indicates the thermal conductivity of PVDF/CNT thin film. Besides, in view of surface thermal radiation and heat convection with the ambient environment under a semi-closed system, two other equations were introduced

3 $${{{{{\boldsymbol{-}}}}}}{{{{{{\boldsymbol{n}}}}}}}_{{{{{{\bf{rad}}}}}}} \cdot {{{{{{\boldsymbol{q}}}}}}}_{{{{{{\bf{rad}}}}}}}=\varepsilon {\sigma }_{{{{{{\rm{SB}}}}}}}\left({T}_{{{{{{\rm{amb}}}}}}}^{4}-{T}^{4}\right),$$

4 $$-{{{{{{\boldsymbol{n}}}}}}}_{{{{{{\bf{conv}}}}}}} \cdot {{{{{{\boldsymbol{q}}}}}}}_{{{{{{\bf{conv}}}}}}}=h\left({T}_{{{{{{\rm{amb}}}}}}}-T\right),$$

where *ε* and *σ*_SB_ refer to the surface emissivity and Stefan-Boltzmann constant, respectively. *T*_amb_ and *T* are the ambient and PVDF-based device temperatures, *h* donates the heat convection coefficient. ${{{{{{\boldsymbol{n}}}}}}}_{{{{{{\bf{rad}}}}}}}$ and ${{{{{{\boldsymbol{q}}}}}}}_{{{{{{\bf{rad}}}}}}}$ represent the thermal radiation vector direction and radiative heat flux, respectively; similarly, ${{{{{{\boldsymbol{n}}}}}}}_{{{{{{\bf{conv}}}}}}}$ and ${{{{{{\boldsymbol{q}}}}}}}_{{{{{{\bf{conv}}}}}}}$ are the heat convection vector direction and convective heat flux. In contrast to uniform heat variation (*M* ≡ 1), the TPG confines the incident solar absorption into a focused hotspot, and the input total thermal power density was tuned by the magnification factor (*M* = *A*/*a* = 71.7).

### Macroscopic mechanism from heat transfer

To understand how the pyroelectric output governed by the macroscopic heat transfer along the transverse and longitudinal directions, we developed a temperature-dependent model by employing the measured device’s parameters. Concerning the macroscopic thermodynamics in the pyroelectric device, the fluctuation in d*T*/d*t* and Δ*T* of the device is expressed as^39,40^

5 $${\eta }_{{{{{{\rm{abs}}}}}}}\left({q}_{{{{{{\rm{i}}}}}}}{aM}\right)={C}_{{{{{{\rm{H}}}}}}}{{{{{\rm{d}}}}}}T/{{{{{\rm{d}}}}}}t+{G}_{{{{{{\rm{T}}}}}}}\Delta T,$$

where ${\eta }_{{{{{{\rm{abs}}}}}}}$ is the solar absorbance (%) of the device, ${q}_{{{{{{\rm{i}}}}}}}$ and *a* are the incident illumination intensity and irradiation area respectively, *C*_H_ and *G*_T_ are the heat capacitance of pyroelectric materials and thermal conductance between the device and sample holder. Considering the device directly contacts with the ambient air (practical measurement), then the *G*_T_ can be derived from Newton’s law of cooling and given as

6 $${G}_{{{{{{\rm{T}}}}}}}={hA}.$$

In view of intermittence time-dependent temperature fluctuations, *C*_H_ can be rewritten to *κA*Δ*t*/*l* for a given small Δ*t* from Fourier’s law of heat conduction at quasi-static conditions (i.e., Δ*T* = d*T* at Δ*t* = d*t*). Consequently, with the introduction of pyroelectric charge from a temperature gradient, i.e., *Q* = *pA*Δ*T*, the Eq. (5) is given by

7 $${\eta }_{{{{{{\rm{abs}}}}}}}({q}_{i}{aM})=\frac{(\kappa /l+h)Q}{p},$$

which is independent of the device area *A*, and the pyroelectric charge is given as

8 $$Q=p({\eta }_{{{{{{\rm{abs}}}}}}}{q}_{i}{aM}){(\kappa /l+h)}^{-1}.$$

Interestingly, the first bracket in Eq. (8) remains stationary since the total absorbed solar power $({\eta }_{{{{{{\rm{abs}}}}}}}{q}_{i}{aM})$ is identical, constant, and *M*-independent for conventional (*A* ≡ *aM* and *M* ≡ 1, i.e., *A* ≡ *a*), as well as thermal ripple induced pyroelectricity (*A* ≡ *aM* and *M* > 1, i.e., *A* > *a* while *A* is constant). These results were verified in the finite element modelling as well (Supplementary Fig. 9f). However, in terms of the second bracket of Eq. (8), i.e., the macroscopic heat transfer, in which the heat propagation/redistribution impacts the local temperature profile and heat conduction length (*l*) at the device, resulting in distinctive *P*_S_ and *Q*. Specifically, the decoupled solar heat confinement and spatial self-propagating thermal ripple fluctuation arising from the quasi-static anomalous conductive heat flux towards thermodynamic disequilibrium. This benefits the in-plane heat perturbation of TPG with a large heat conduction length (*l* = (*w* - *d*_a_)/2, *w* is the width of the device), thus facilitating the boosted surface charge density in the local irradiation area (*a*). By contrast, the solar heat dissipates simultaneously and uniformly at the entire conventional device (*A*) following the out-of-plane direction with a slight heat conduction length (*d*_0_, the device thickness) and relatively low polarization. In other words, the pyroelectric charges of conventional and TPG devices are modulated by a distinctive heat transfer diagram, and the harvested charge under corresponding heat conduction lengths can be written as

9 $$Q=\left\{\begin{array}{c}p\cdot {\eta }_{{{{{{\rm{abs}}}}}}}{q}_{{{{{{\rm{i}}}}}}}A\cdot {\left(\frac{\kappa }{l}+h\right)}^{-1},\left(l={d}_{0},\,{Conventional}\right)\, \\ \!\!\!\!\!\!\!p\cdot {\eta }_{{{{{{\rm{abs}}}}}}}\left({q}_{{{{{{\rm{i}}}}}}}M\right)a\cdot {\left(\frac{\kappa }{l}+h\right)}^{-1},\left(l=\frac{w-{d}_{{{{{{\rm{a}}}}}}}}{2},{TPG}\right)\end{array},\right.$$

in which the *Q* of the conventional device is proportional to solar intensity and device area while independent of *M*, this is the generality of conventional pyroelectrics, where the input solar heat is coupled with the heat delivery in the entire device^37^. In contrast, the overall solar illumination is governed by *M* and incident intensity (${q}_{{{{{{\rm{i}}}}}}}$), leading to an upgrading pyroelectric output as the *M* nonlinearly varies with the illumination area (*a*) (Supplementary Fig. 12c), although the total received solar power is identical to the conventional design.

### Lattice dynamics of transverse pyroelectricity

On the other hand, the focused quasi-static conductive heat flux and temperature-dependent $p$ at the confined irradiation area are enlarged synergistically by the tuneable magnification for thermal ripple mediated pyroelectrics (Fig. 4c). To uncover the in-depth microscopic fundamentals of transverse pyroelectricity (i.e., the origin of polarization and pyroelectricity ($p$ in Eq. (8)), and the temperature dependence behaviours) beyond above-mentioned macroscopic understandings, we provided additional discussion based upon lattice dynamics and thermodynamics of pyroelectricity. According to the phenomenological theory of the free energy of a lattice^35^, the electric polarization (*P*) is the “force” conjugate to the known “thermal electric field” (-*E*), so the observed *P* under a finite temperature (*T*) can be obtained from the derivative of free energy (*F*) of unit volume of a free crystal with respect to -*E*

10 $${P}_{{{{{{\rm{S}}}}}}}\left(T\right)=-{\left(\frac{\partial F}{\partial E}\right)}_{{{{{{\rm{T}}}}}}}.$$

Using Eq. (10), the primary pyroelectric coefficient (under external constant strain but internal vibrating lattice, i.e., “clamped-lattice” pyroelectricity, in which the pyroelectricity associated with external thermal expansion/deformation induced piezoelectricity is negligible) is directly governed by the lattice dynamics and can be written by

11 $$p\left(T\right)=\frac{\partial {P}_{{{{{{\rm{S}}}}}}}}{\partial T}.$$

To find the origin of primary pyroelectric effect and its temperature-dependent variation (initially discussed by Boguslawski in 1914 and named “monochromatic” theory), tremendous efforts have been made during the past century. Based on the understanding of lattice dynamical theory, Born had indicated that the primary pyroelectricity in the ion crystals originates from the harmonic variation of ions, in which the electronic renormalization (when d*T*/d*t* > 0) owing to thermal vibrations (i.e., electron-phonon renormalization) with respect to the original state (when d*T*/d*t* = 0), and the dipole moment contribution is proportional to the mean square amplitude of oscillations^30^. However, the role of mechanical and electrical anharmonicity in primary pyroelectricity was thought as “secondary important” in Born’s discussion. Thirty years later, in 1975, Szigeti complemented the anharmonic potential (i.e., the mean displacement of atoms with respect to rigid ions, also known as the internal strain term) into Born’s solution and proved its nonnegligible contribution to primary pyroelectricity^34^. Therefore, in accordance with Born-Szigeti theory of pyroelectricity and expanding the ${P}_{{{{{{\rm{S}}}}}}}\left(T\right)$ with respect to the normal mode amplitudes ($\xi$), the overall primary pyroelectric coefficient can be given as

12 $$p\left(T\right)={p}_{1}+{p}_{2}=\mathop{\sum }\limits_{a}\frac{\partial {P}_{{{{{{\rm{S}}}}}}}}{\partial {\xi }_{a}}\frac{d\left\langle {\xi }_{a}\right\rangle }{{dT}}+\mathop{\sum }\limits_{j}\frac{{\partial }^{2}{P}_{{{{{{\rm{S}}}}}}}}{\partial {\xi }_{j}^{2}}\frac{d\left\langle {\xi }_{j}^{2}\right\rangle }{{dT}},$$

where ${p}_{1}$ is the first-order primary coefficient ascribed to rigid-ion contribution (induced by the mean displacement of ions carrying Born effective charge), ${p}_{2}$ is the second-order primary coefficient owing to electron-phonon renormalization (i.e., Debye-Waller term). In the second term (${p}_{2}$) of the right-hand side of Eq. (12), the mean square displacement associated with all phonon modes and can be expressed by

13 $$\left\langle {\xi }_{j}^{2}\right\rangle=\frac{{{\hslash }}}{2}\frac{2{n}_{j}+1}{{\omega }_{j}},$$

in which ${\omega }_{j}$ and ${n}_{j}$ refer to the eigenfrequency and the Bose-Einstein distribution, respectively. For a simple estimation, the ${\omega }_{j}$ can be represented by using phonon frequency. Here, in terms of transverse pyroelectricity, the microscopic mechanism of how the achieved giant performance associated with the primary pyroelectric coefficient that increases under rising temperatures can be ascribed to the temperature dependence of $\langle {\xi }_{a}\rangle$ and/or $\langle {\xi }_{j}^{2}\rangle$. In recent years, the contribution of electron-phonon renormalization (${p}_{2}$) to pyroelectricity has been overlooked as negligible in traditional views, is found to be significant^2^. In particular, it is revealed that the $\langle {\xi }_{j}^{2}\rangle$ increased linearly with respect to temperature owing to decreasing frequency of phonons of thick and thin films in various material systems^2,5,31^. This conclusion is applicable to proposed transverse pyroelectricity, in which a higher temperature benefits to increase $\langle {\xi }_{j}^{2}\rangle$ that contributed from optical modes and therefore improving the ${p}_{2}$, especially for non-uniform polarization^33^. Meanwhile, the unprecedented temperature ripple travelling along the transverse direction in TPG offers nonnegligible internal strain (e.g., owing to the variation of unit cell volume^31^, the rotation of the C-F and C-H bonds in β-PVDF with temperatures^29^), thus enabling nonvanishing atomic displacement with respect to ions to contribute the ${p}_{1}$. Aside from that, the rigid-ion associated with electron-phonon renormalization and external thermal expansion may play a possible role in the increment of overall transverse pyroelectricity. Although the experimental quantification of how ${p}_{1}$ and/or ${p}_{2}$ dominates the heat-induced polarization ripple change is still changeable (e.g., anharmonic and harmonic phonon dispersion relations and their temperature dependence, lattice dimensionality, dopant polarizability), the above discussions pave a possible way to understand the lattice dynamics with inhomogeneous heat manipulation and could be of great interests in the future.

### Outdoor test of scalable TPG systems

The outdoor test was conducted at the location of 22°36'30'' N, 113°59'43'' E, the ground behind Block 8, Lychee Hills, Southern University of Science and Technology, with a time duration of 7.30 AM to 5.30 PM, June 16, 2021. The scalable TPG systems (9 units, in parallel) were arranged under tree shadow, and the incident sunlight from wind-driven tree shadow fluctuation facilitated the hotspot variation on the TPG surface. The electrical signal of TPG systems was recorded using a 6514 electrometer. The TPG temperature was monitored at the central position of the TPG system by a temperature datalogger (SSN-61, YUWESE). The irradiation intensity variation was measured using a solar power meter datalogger (1333 R, TES). The ambient temperature, ambient relative humidity, and wind speed were recorded by a hot-wire anemometer (1341, TES). All data were collected simultaneously and connected to a laptop for processing.

## Supplementary information

Supplementary Information

Description of Additional Supplementary Files

Supplementary Movie 1

## Data Availability

The datasets generated during and/or analyzed during the current study are available from the corresponding author on reasonable request.

## Code availability

The code utilized during the current study is available from the corresponding author on reasonable request.
