# Supplementary material for: Giant polarization ripple in transverse pyroelectricity
Source: Nat Commun. 2023 Jan 26;14:426. doi: 10.1038/s41467-023-35900-x (PMC9879950; doi:10.1038/s41467-023-35900-x)
Supplement: Supplementary file 1 — Supplementary Information [file 41467_2023_35900_MOESM1_ESM.pdf]

Supplementary Information for

Giant polarization ripple in transverse pyroelectricity

Yi Zhou[1,2,7], Tianpeng Ding[1,3,7], Jun Guo[1], Guoqiang Xu[1], Mingqiang Cheng[4], Chen Zhang[1], Xiao-Qiao Wang[1], Wanheng Lu[1], Wei Li Ong[1], Jiangyu Li[4], Jiaqing He[2,*], Cheng-Wei Qiu[1,*] and Ghim Wei Ho[1,5,6,*]

[1]Department of Electrical and Computer Engineering, National University of Singapore, Singapore, Singapore.

[2]Department of Physics, Southern University of Science and Technology, Shenzhen, China.

[3]School of Electronic Science and Engineering, State Key Laboratory of Electronic Thin Film and Integrated Devices, University of Electronic Science and Technology of China, Chengdu, China

[4]Department of Materials Science and Engineering, Southern University of Science and Technology, Shenzhen, China.

[5]Department of Materials Science and Engineering, National University of Singapore, Singapore, Singapore.

[6]Institute of Materials Research and Engineering, A*STAR (Agency for Science, Technology and Research), Singapore, Singapore.

[7]These authors contributed equally: Yi Zhou, Tianpeng Ding.

*E-mail addresses: hejq@sustech.edu.cn; chengwei.qiu@nus.edu.sg; elehgw@nus.edu.sg

This supplement contains:

Supplementary Notes 1-5

Supplementary Figures 1-24

Supplementary Tables 1-3

Supplementary Movie 1

Supplementary References

Table of Contents

Supplementary Note 1. Extraction of the pyroelectric coefficient 4

Supplementary Note 2. Discussion on the anisotropic polarization in pyroelectrics 5

Supplementary Note 3. Measurement and discussion of non-primary pyroelectricity 7

Supplementary Note 4. Load measurement of modular conventional and TPG systems 8

Supplementary Note 5. Calculation of peak power output 9

Supplementary Fig. 1 Thermal and electrical measurement configuration of pyroelectricity. 10

Supplementary Fig. 2 Temporal temperature-induced electrostatic process of pyroelectricity and solar-thermal characterization. 11

Supplementary Fig. 3 Measured pyroelectric coefficient, temperature, and polarization profiles of PVDF-based conventional device. 12

Supplementary Fig. 4 Schematic of longitudinal and transverse polarization measurement... 13

Supplementary Fig. 5 Output of TPG and piezoelectric device for indication of negligible non-primary pyroelectricity. 14

Supplementary Fig. 6 Temporal current characteristics of conventional and TPG devices under light on/off conditions. 15

Supplementary Fig. 7 Temporal voltage characteristics of conventional and TPG devices under light on/off conditions. 16

Supplementary Fig. 8 Geometries for finite element analysis of heat transfer. 17

Supplementary Fig. 9 Finite element analysis of heat transfer for conventional and TPG devices 18

Supplementary Fig. 10 Finite element analysis of electrical output for conventional and TPG devices. 19

Supplementary Fig. 11 Electrical output of conventional and TPG devices at periodic heating/cooling process. 20

Supplementary Fig. 12 Light illumination area and optical profiles 21

Supplementary Fig. 13 Full-parameter optimization of the electrical output of TPG 22

Supplementary Fig. 14 Current-voltage characteristics of pyroelectric output. 23

Supplementary Fig. 15 Enhanced output of size scaling down TPG. 24

Supplementary Fig. 16 Enhanced output of TPG using inorganic ceramics 25

Supplementary Fig. 17 Enhanced output of TPG using asymmetrical solar absorbers with distinctive inhomogeneous thermal conductivities (κ-TPG) 26

Supplementary Fig. 18 Conventional and TPG systems for the outdoor test.. 27

Supplementary Fig. 19 Load characteristics and output power of scalable conventional and TPG systems. 28

Supplementary Fig. 20 Enhanced TPG using multiple transverse heat ripples. 29

Supplementary Fig. 21 Peak power density of conventional and TPG devices versus illumination intensities 30

Supplementary Fig. 22 Comparison of power output of ferrophotovoltaics, conventional pyroelectrics, and proposed TPG device. 31

Supplementary Fig. 23 Measurement of solar thermoelectric generation under heating/cooling process. 32

Supplementary Fig. 24 Outdoor-under-tree-shadow test of scalable TPG system. 33

Supplementary Table 1. Parameters for conventional and transverse pyroelectricity simulations 34

Supplementary Table 2. Calculation of gain factors for conventional and TPG devices 35

Supplementary Table 3. State-of-the-art comparison of solar pyroelectrics, ferrophotovoltaics, and thermoelectrics 36

Supplementary Movie 1. Outdoor test of transverse pyroelectric generation systems 37

Supplemental References 38

Supplementary Note 1. Extraction of the pyroelectric coefficient

In terms of temperature-dependent pyroelectric coefficient measurement, the Byer-Roundy method was introduced for dynamic time-dependent temperature $T(t)$ and current $I(t)$ characteristics identification[1-5]. The pyroelectric current formula, $I = \mathrm{d}Q/\mathrm{d}t = p \cdot a \cdot \mathrm{d}T(t)/\mathrm{d}t$ gives the pyroelectric coefficient as

$$p = \frac{I(t)}{a \cdot \mathrm{d}T(t)/\mathrm{d}t} \tag{S1}$$

where the generated current $I(t)$ is dominated by the primary pyroelectric effect, and p (T) can be obtained from a small temperature interval under an illumination area a. Specifically, a pyroelectric device was illuminated under periodic heating/cooling cycles at varied solar irradiation intensities from 2.5 to 15.0 mW cm^{-2} with an interval of 2.5 mW cm^{-2}. The electric current and temperature were recorded simultaneously for p (T) derivation (Supplementary Figs. 1-3, details can be found in Methods). Moreover, governed by Fourier's law of heat conduction, the quasi-static conductive heat flux ($\boldsymbol{q}_{\mathrm{cond}}$) of the device was estimated using

$$-\boldsymbol{n}_{\mathrm{cond}} \cdot \boldsymbol{q}_{\mathrm{cond}} = \frac{\kappa \cdot A \cdot \Delta T}{l} \tag{S2}$$

where ΔT stands for temperature amplitude/difference, κ and A represent the thermal conductivity and area of the device, respectively; l refers to the heat conduction length corresponding to the heat flux vector direction $\boldsymbol{n}_{\mathrm{cond}}$. For conventional (CONV) out-of-plane heat variation, where l equals the device thickness (d_0), and ΔT denotes the relatively small temperature difference (< 1°C) between the top and bottom surfaces/electrodes. In contrast, the modulated solar heat at the illuminated hotspot redistributes the heat conduction, leading to an in-plane/transverse heat perturbation, where ΔT is ascribed to the central and edge temperatures. The l is modified to the distance between the illuminated hotspot and edge, i.e., $l = (w - d_a)/2$, where w and d_a represent the width of the device and the diameter of the hotspot, respectively.

Supplementary Note 2. Discussion on the anisotropic polarization in pyroelectrics

The anisotropic nature of pyroelectric materials offers a different polarization along the longitudinal and transverse directions. Indeed, the spontaneous polarization (Ps, electric dipole moment per unit volume) in polar materials originally lies in the domain orientation (the degree of structure freedom along the crystallographic orientation) and domain wall densities, which has been experimentally examined in single crystals and polycrystalline during the past decade[6-8]. Also, the anisotropic polarization of pyroelectric films can be manipulated at different crystallographic orientations via strain engineering owing to their non-centrosymmetric crystal structures[9,10]. On the other hand, the reported anisotropic Ps in pyroelectric thin films not only depends on crystal orientation but the electrodes' location [11,12]. Normally, the longitudinal Ps is measured via a parallel-plate capacitor, in which top and bottom electrodes are utilized (Supplementary Fig. 4a). By contrast, the coplanar interdigital capacitor with one side deposited two different comb-like finger electrodes is utilized for transverse Ps measurement (Supplementary Fig. 4b). In this work, the device configuration used for conventional (Fig. 1a) and transverse pyroelectric (Fig. 1b) measurement is identical, i.e., a parallel-plate capacitor with a sandwiched structure of electrode/pyroelectric/electrode (PEDOT/PVDF/PEDOT) (Supplementary Fig. 4a). The solar absorber CNT was coated on the upper side of the PEDOT layer, and copper wires were used to connect the upper and bottom PEDOT electrodes to the testing instruments (Supplementary Fig. 2, TPG fabrication and characterization in Methods). The pyroelectric intensity (temperature-differential polarization) was indirectly measured via a parallel-plate capacitor (Supplementary Fig. 4a, Supplementary Note 1). Therefore, the pyroelectric intensity is the sum value (i.e., Ps is the net polarization), and other non-primary pyroelectric effects are negligible (Supplementary Note 3, Supplementary Fig. 5). As known the ΔPs is governed by the temporal temperature change, so we specified the non-uniform net polarization ripple with respect to heat ripples in Fig. 1b, which is different from the uniform

conventional polarization diagram (Fig. 1a and Supplementary Fig. 2a). More importantly, in accordance with pyroelectric fundamentals, the nonlinear characteristics of temperature-dependent ΔP_S (electric dipole moment change) varies slightly at low temperature while significantly when approaching the Curie temperature (T_{Curie})[4,13,14]. In other words, an intense heat variation (dT/dt) from a large, graded temperature gradient (ΔT) is capable of creating a giant polarization ripple (i.e., ΔP_S or larger dipole moment shift, θ), attributed to higher electrostatic intensity induced surface charge density (σ). Therefore, we primarily focused on how the facile, macroscopic, in-plane heat manipulation impacts the polarization ripple propagation and corresponding pyroelectric harvesting performance in the absence of tailoring materials properties[15,16], altering pyroelectric coefficients[4,17,18], or applying electric filed[19,20]. We solely confined the incident light onto the PVDF-based polar film to trigger non-uniform heat ripple traveling from the hotspot to non-irradiated areas along the in-plane direction (Fig. 1b), thus achieving intense non-static dT/dt and heat-induced P_S changes. Unlike conventional pyroelectricity (Fig. 1a), where the P_S and voltage potential are dominated by the slight out-of-plane heat conversion and dissipation across the entire bulk device. Herein we aim to systematically investigate the heat-induced polarization ripple in the transverse pyroelectricity and verify its priority and effectiveness for boosted heat harvesting performance, compared with traditional longitudinal configurations. Aside from that, we believe the manipulation of anisotropic properties (e.g., electric polarizations, thermal conductivities, absorbances) of pyroelectrics from the material view (e.g., controllable domain wall orientation and/or densities via strain engineering, oriented polarization manipulation via electric polling, epitaxial growth of heterophase) is promising in heat harvesting, and which could be of great interests and improve the transverse pyroelectricity greatly in the future.

Supplementary Note 3. Measurement and discussion of non-primary pyroelectricity

The solar heat confinement in the illuminated area of TPG device may lead to thermal stress-induced piezoelectric output and polarization gradient in ferroelectric PVDF thin films[21-23]. From a view of non-uniform heat distribution induced thermal expansion (Δd_t) of TPG, i.e., $\Delta d_t = d_0(\mu\Delta T)$, where μ is the thermal expansion coefficient of PVDF thin film, and d_0 refers to the device thickness[24], thereby the thermal expansion at the thickness direction is estimated to be around 6 μm at 10 mW cm^{-2} illumination for TPG (Fig. 2a, Supplementary Fig. 2c). To clarify the as-mentioned non-primary pyroelectric effects in the TPG output, a PVDF-based device was utilized for contact/separation (simulated thermal stress) strain-induced piezoelectric measurement (Supplementary Fig. 5). The in-plane geometric centre position of the stationary piezoelectric device was contacted by a moving probe (probe diameter equals d_a, i.e., 4 mm) with a speed of 1.0 mm s^{-1}. The initial distance between the stationary piezoelectric device and the probe was around 30 mm. The contact/separation duration ratio was fixed at 0.5, similar to the heating/cooling time ratio for TPG measurement. The measured deformation (supposed thermal expansion Δd_s) at the centre position of the piezoelectric device was around 550 μm (far greater than Δd_t = 6 μm). Moreover, the piezoelectric voltage and current were recorded by a 6514 electrometer under periodic contact/separation cycles, and the whole piezoelectric test was conducted in a Faraday cage to minimize the noise. The piezoelectric output at an equivalent thermal deformation of 6 μm was plotted in Supplementary Fig. 5, according to piezoelectric governing equations[24-26].

Supplementary Note 4. Load measurement of modular conventional and TPG systems

The load measurement of modular conventional and TPG systems was conducted at an illumination intensity of 10.0 mW cm^{-2} with a heating/cooling (light on/off) time duration ratio of 0.5. A tunable load resistor (ZX79, Fuyang Precision) serially connected with modular systems was used to vary the matched impedance and load current. For modular systems in parallel measurement, the load resistance was switched at 1, 10, 50, 100, 500, 1000, 2500, 5000, and 10000 (unit: MΩ), separately. Similarly, the load resistance for modular systems in series was tuned from 0.1, 0.5, 1.0, 5.0, 10.0, 25.0, 50.0, and 100.0 (unit: GΩ). The corresponding current signal was recorded using a 6514 electrometer under ten periodic heating/cooling cycles for each load resistance. Based upon Kirchhoff's law of current and Ohm's law, the power (P_{e}) was extracted by time integration of current under corresponding load impedance (R)[27,28]

$$P_{\mathrm{e}} = \frac{1}{t}\int_{t_0}^{t_0+t} I^2 R t \mathrm{d}t \tag{S3}$$

where the initial time is t_0, the integration time t was selected from the recorded time-dependent current signals. The calculated results using equation (S3) is the average power in the time duration t, and not the peak power.

Supplementary Note 5. Calculation of peak power output

The peak power output of the device is dependent on the maximum current, voltage profiles, internal resistance, and load impedance. Typically, the peak power ($P_{\max}$) of a single device is extracted from time-dependent current or voltage profiles under a matched load resistance evaluated under transient temperature fluctuations. In specific, the $P_{\max}$ is achieved as long as the internal resistance of the device equals the matched load resistance[27,29], as given by

$$P_{\max} = \frac{I_{\max} \cdot V_{\max}}{4} \tag{S4}$$

where $I_{\max}$ and $V_{\max}$ represent the peak-to-peak current (short-circuit) and voltage (open-circuit) calculated from time-dependent signals at multiple heating/cooling cycles, respectively. Correspondingly, the Carnot efficiency ($\eta_{\text{Carnot}} = (T_{\text{h}}\text{-}T_{\text{l}})/T_{\text{h}}$, where T_{h} and T_{l} are the high and low temperature per heating/cooling cycle for the illuminated area of a device) and the energy conversion efficiency of solar pyroelectric generation is evaluated by the $P_{\max}$ and incident intensity (q_{solar})

$$\eta_{\max} = \frac{P_{\max}}{q_{\text{solar}}} \tag{S5}$$

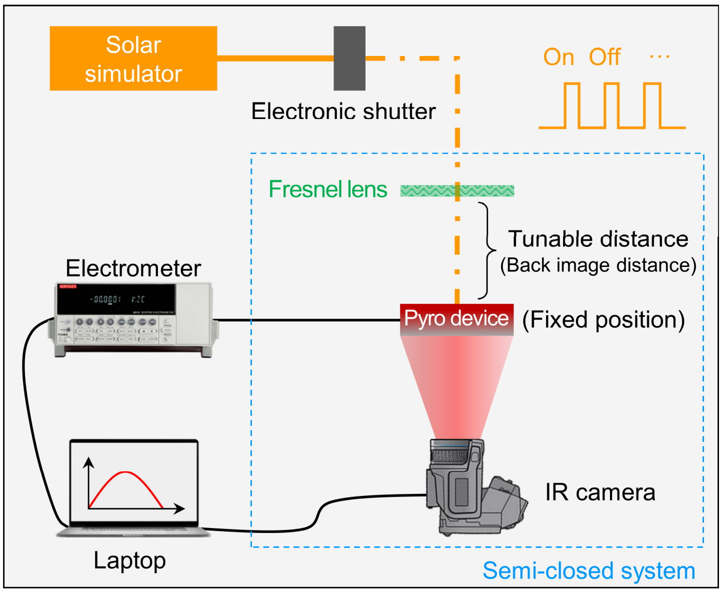

Supplementary Fig. 1 Thermal and electrical measurement configuration of pyroelectricity. A solar heat manipulator (Fresnel lens), pyroelectric device, and infrared camera were vertically positioned in a semi-closed system with a controlled ambient temperature (28°C) and relative humidity (60%). The lens-device distance (back image distance) was adjustable to allow manipulation of light magnification and solar heat redistribution onto the device. Besides, the on/off (heating/cooling) ratio for the electronic shutter was fixed at 0.5 (light on, 30 seconds; light off, 60 seconds). The electronic shutter, electrometer, and IR camera were simultaneously connected to a laptop for data acquisition. The inset photos were taken/drawn by the authors.

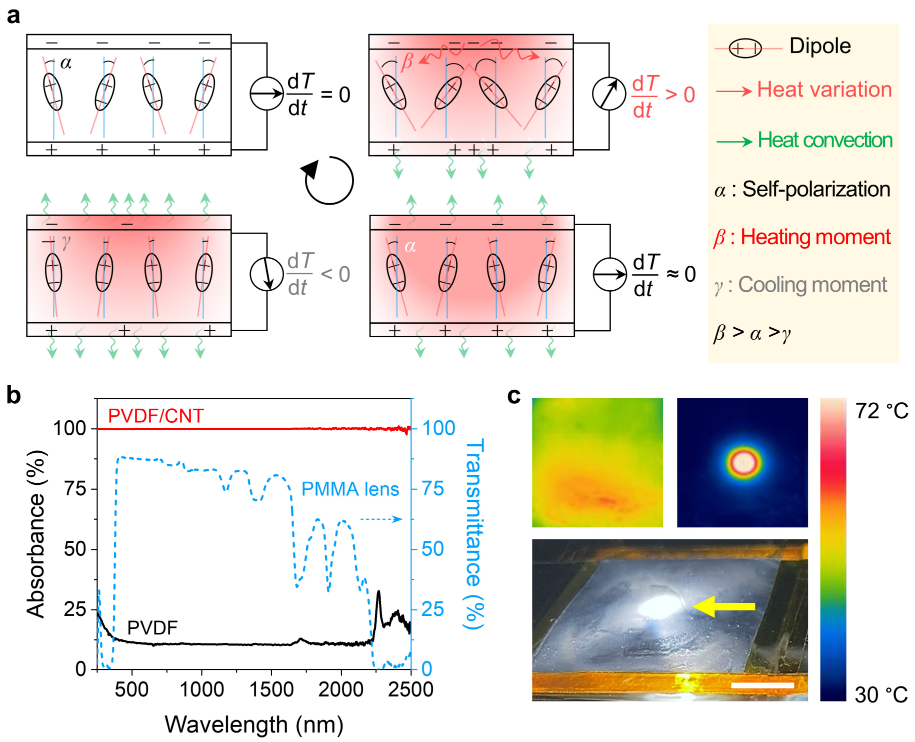

Supplementary Fig. 2 Temporal temperature-induced electrostatic process of pyroelectricity and solar-thermal characterization. (a) Diploe moment changes and heat diagram of conventional intrinsic polarization under uniform heating/cooling conditions, where α, β, γ are dipole moment shift angles at thermal-equilibrium, heating, and cooling conditions, respectively. The relationship $\beta > \alpha > \gamma$ is applicable for the device under uniform heat variation. **(b)** Absorbance (solid lines on the left side) and transmittance (dashed line on the right side) of PVDF/CNT, PVDF, and PMMA lens. **(c)** Infrared images of conventional (upper left) and TPG (upper right) at 10 mW cm^{-2} illumination. Photographic image (lower) of TPG with a localized hotspot (yellow arrow) under irradiation. Scale bar: 1 cm.

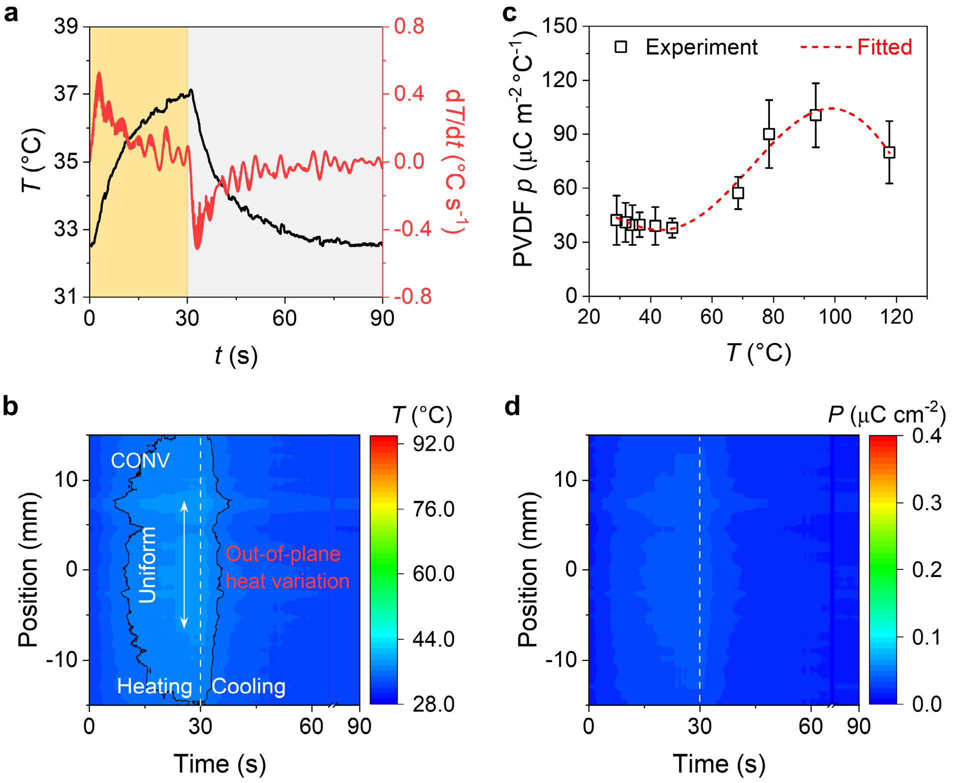

Supplementary Fig. 3 Measured pyroelectric coefficient, temperature, and polarization profiles of PVDF-based conventional device. (a) Average temperature and dT/dt extracted from the device surface. **(b)** Measured spatial and temporal temperature distributions under a uniform heating/cooling process. **(c)** Pyroelectric coefficient of PVDF-based device versus temperature. The fitted formula is $p(T)$ = 1.2239E-7T^5 - 4.68522E-5T^4 + 6.16E-3T^3 - 0.32908T^2 + 6.91557T, where R-Square (COD) = 1. **(d)** Measured spatial and temporal polarization distributions of the device under uniform heating/cooling process. The illumination is 10 mW cm^{-2}.

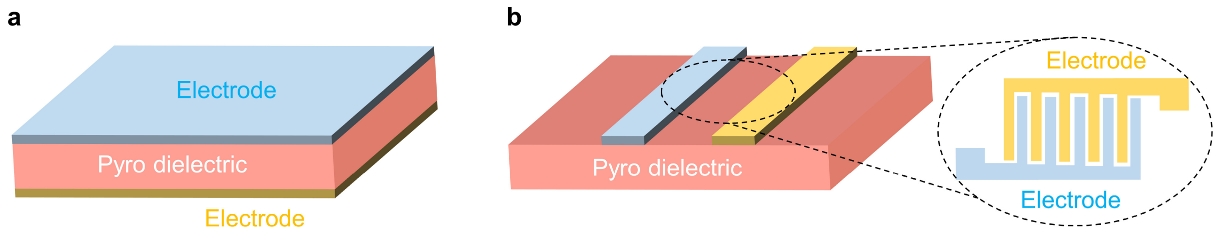

Supplementary Fig. 4 Schematic of (a) parallel-plate capacitor for measuring longitudinal polarization and (b) coplanar interdigital capacitor for transverse polarization measurement. In this work, the device configuration (a) was utilized for the pyroelectric coefficient (temperature differential of polarization) measurement of PVDF thin film. All the measurements for conventional and TPG devices were performed by using an identical sample (details can be found in the Methods and Supplementary Note 1). The anisotropic polarization was not considered in this measurement, and the measured pyroelectric coefficient was the net value (mainly ascribed to the primary pyroelectric effect).

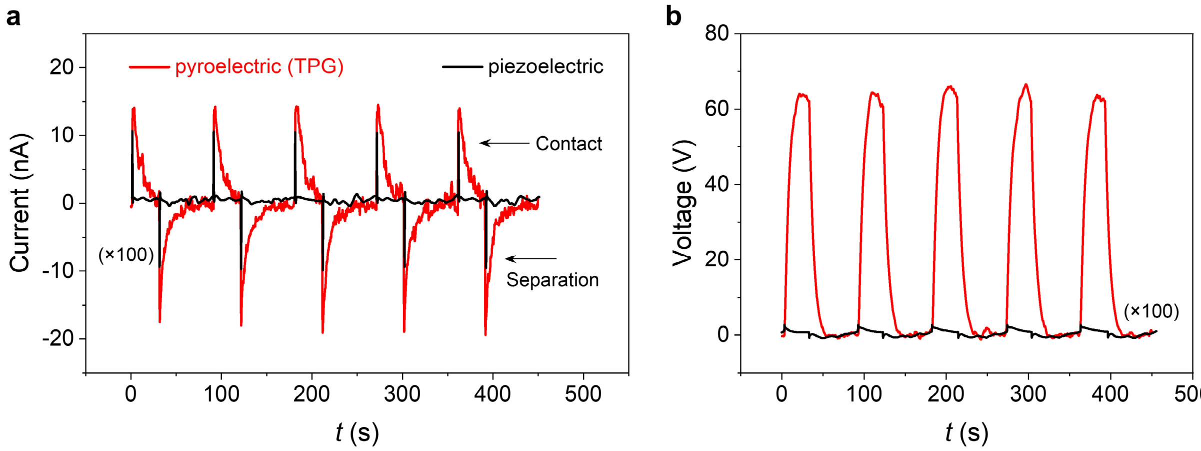

Supplementary Fig. 5 Output of TPG and piezoelectric device for indication of negligible non-primary pyroelectricity. (a) Current and **(b)** voltage fluctuations under the light on/off for TPG at 10 mW cm^{-2} illumination, and contact/separation for a piezoelectric device (equivalent thermal deformation of ~ 6 μm). The piezoelectric output was enlarged by ×100 for comparison. The whole test was conducted using a single device. The time duration ratio for the light on/off (pyroelectric) and the contact/separation (piezoelectric) were fixed at 0.5.

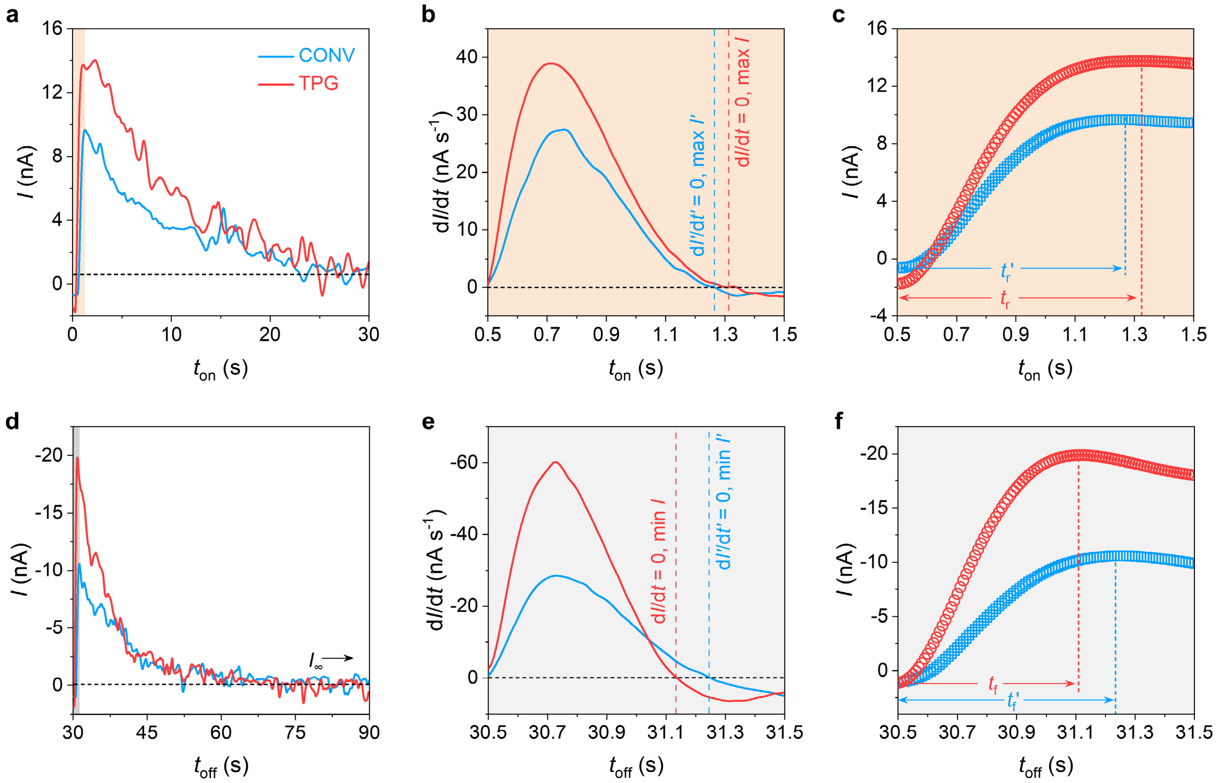

Supplementary Fig. 6 Temporal current characteristics of conventional and TPG devices under light on/off conditions. (a) Current profiles at light on (heating) process. **(b)** Current differential values and **(c)** extraction of rising time (t_r). **(d)** Current profiles at the light off (cooling) process. **(e)** Current differential values and **(f)** extraction of falling time (t_f). The time of peaked maximum and minimum current were extracted at dI/dt = 0, and the rising, as well as falling time, can be evaluated. The illumination is 10 mW cm^{-2}.

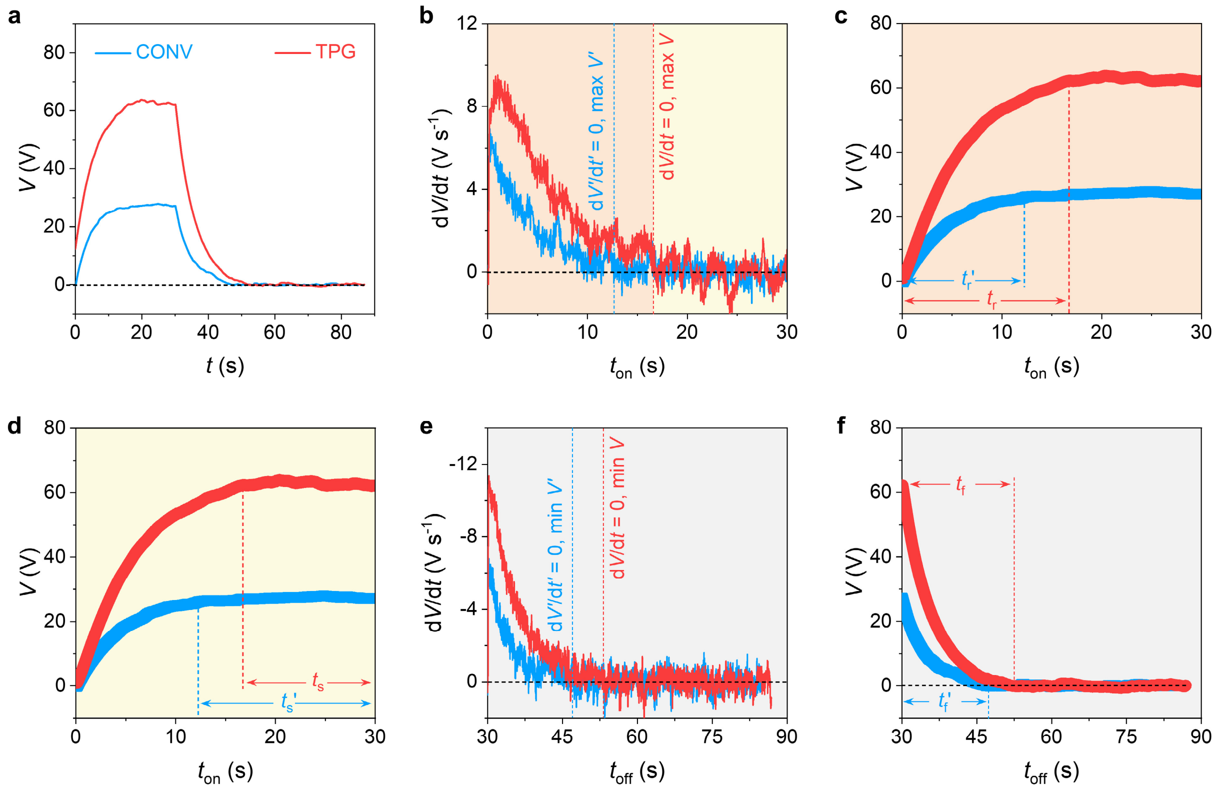

Supplementary Fig. 7 Temporal voltage characteristics of conventional and TPG devices under light on/off conditions. (a) Voltage profiles versus time. **(b)** Voltage differential values versus time. **(c-d)** Extraction of rising (t_r) and saturation time (t_s) under heating process. **(e)** Voltage differential values and **(f)** extraction of falling time (t_f) under the cooling process. The time of peaked maximum and minimum voltage was extracted at dV/dt = 0, and the rising, saturation, as well as falling time can be evaluated. The illumination is 10 mW cm^{-2}.

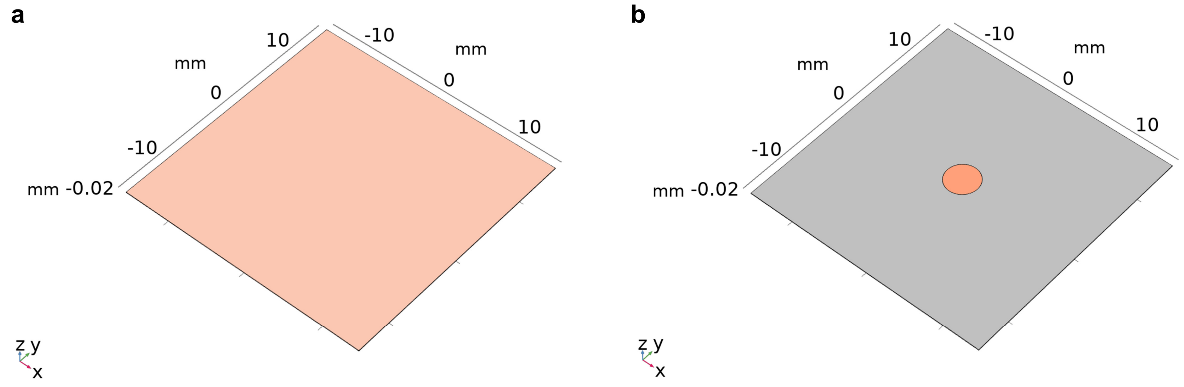

Supplementary Fig. 8 Geometries for finite element analysis of heat transfer. (a) Conventional and **(b)** TPG devices. The orange area stands for the illumination area.

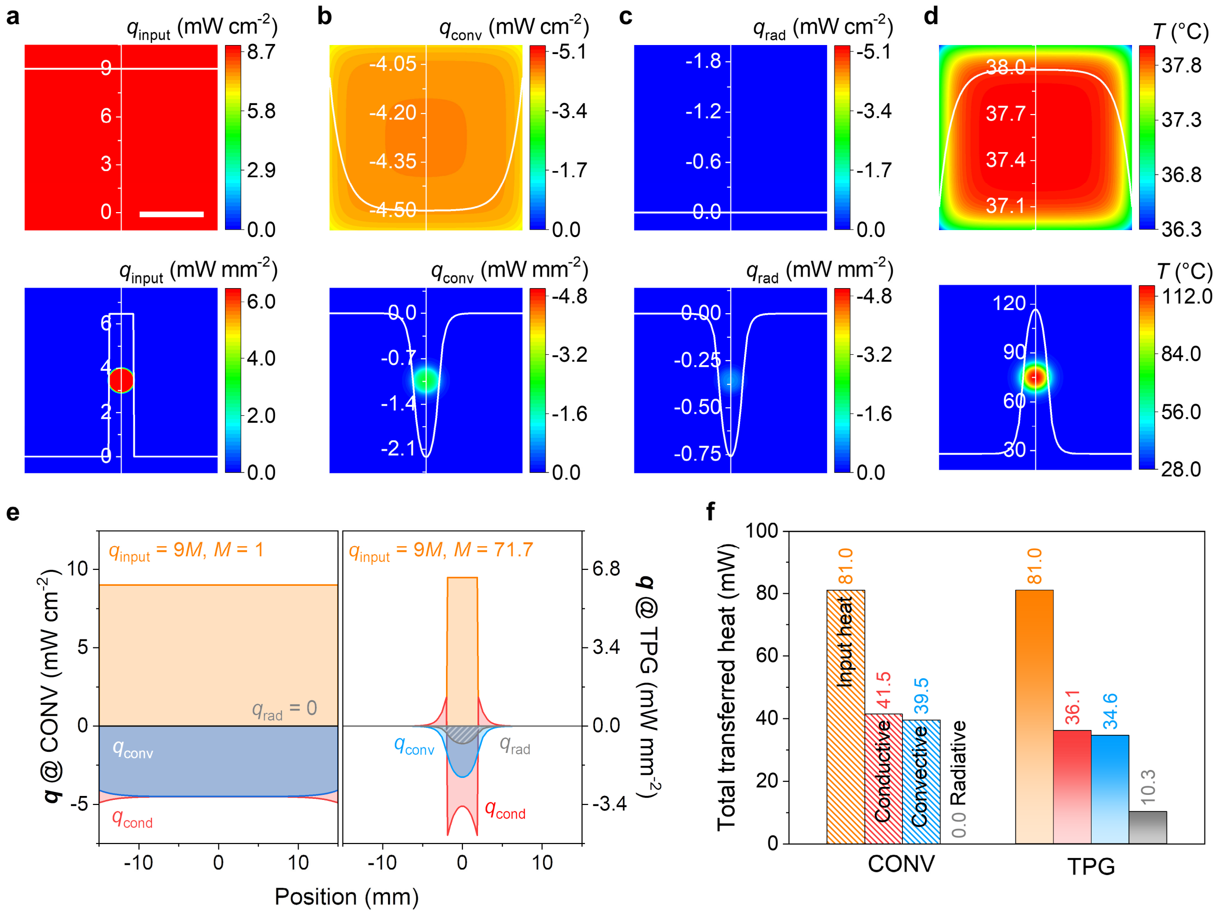

Supplementary Fig. 9 Finite element analysis of heat transfer for conventional and TPG devices. **(a)** Input heat flux, **(b)** quasi-static conductive heat flux, **(c)** quasi-static radiative heat flux and **(d)** temperature. Upper rows, conventional device; lower rows, TPG. Scale bar: 10 mm. **(e)** Estimated heat flux diagram and **(f)** total transferred heat of conventional and TPG devices. The *M* specifies the magnification of the device area versus the illumination area.

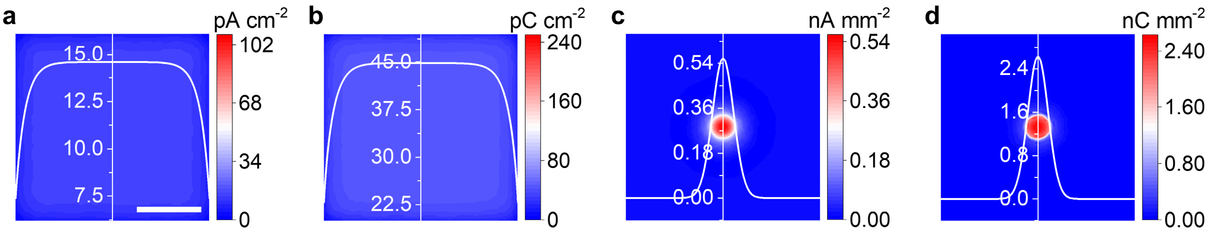

e

Pyroelectric input & output parameters	CONV	TPG	Ratio	Extracted methods
Input solar intensity, q_{solar}	10 mW cm^{-2}		1.0	/
Magnification, M	1	71.7	71.7	/
Input heat flux, q_{input} (input heat)	$9M$ mW cm^{-2} (81 mW)		1.0	Absorbance of 0.9
Conductive heat flux, q_{cond} (mW cm^{-2})	5.1	5.5	1.1	Mean values (area A)
Conductive solar heat (mW)	41.5	36.1	0.9	2D Integrate (area A)
Quasi-static heat flux, $q_{in\text{-}plane}$ (mW cm^{-2})	5.1	84.7	16.6	Normalized (irradiation area)
Polarization, P (μC cm^{-2})	0.3	1.1	3.7	2D Integrate (area A)
V (V)	30.1	99.3	3.3	2D Integrate (area A)
Q (nC)	72.9	158.3	2.2	2D Integrate (area A)
I (nA)	23.7	41.9	1.8	2D Integrate (area A)

Supplementary Fig. 10 Finite element analysis of electrical output for conventional and TPG devices. Current and charge density for **(a-b)** conventional and **(c-d)** TPG. Scale bar: 10 mm. **(e)** Estimated enhancement ratios of TPG versus conventional device. "2D Integrate" stands for double integral in the x-y plane, and the integral area is device area A; "Normalized" represents the mean value at the illumination area a.

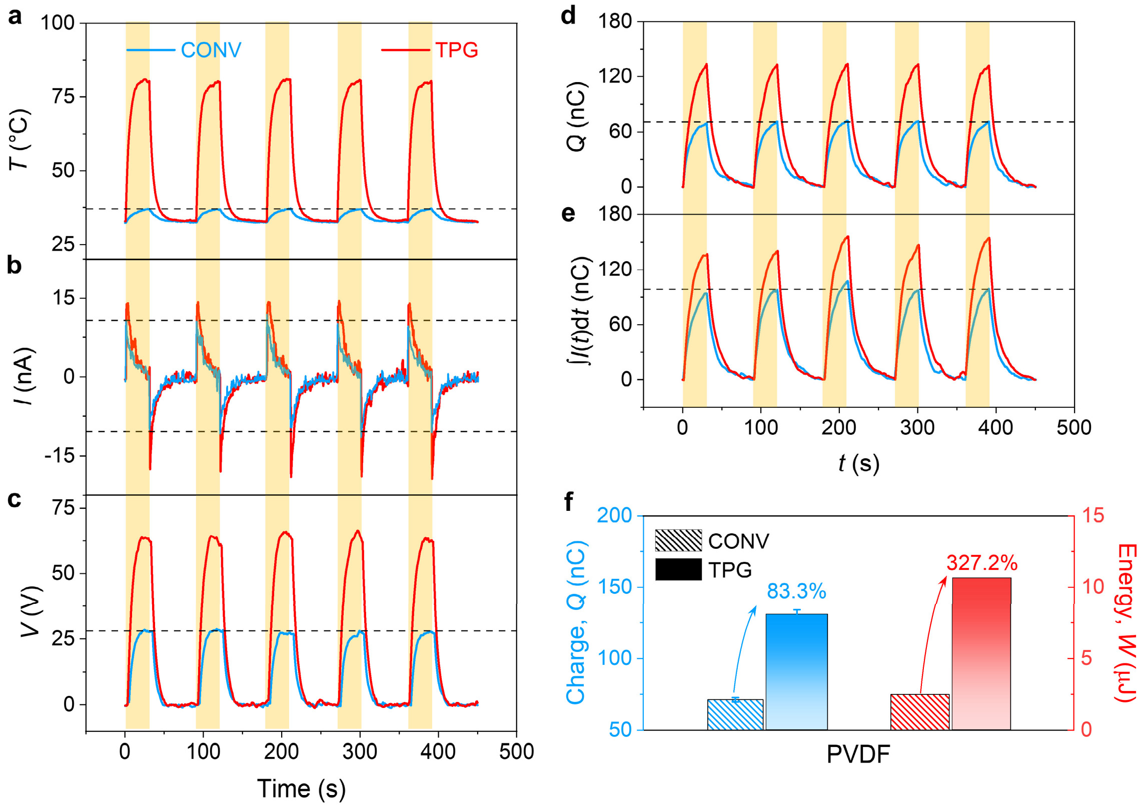

Supplementary Fig. 11 Electrical output of conventional and TPG devices at periodic heating/cooling process. (a) Temperature, **(b)** current, **(c)** voltage, **(d)** charge variations, and **(e)** charge variation integrated from the current profile. **(f)** Comparison of charge and harvested energy. The illumination is 10 mW cm^{-2}.

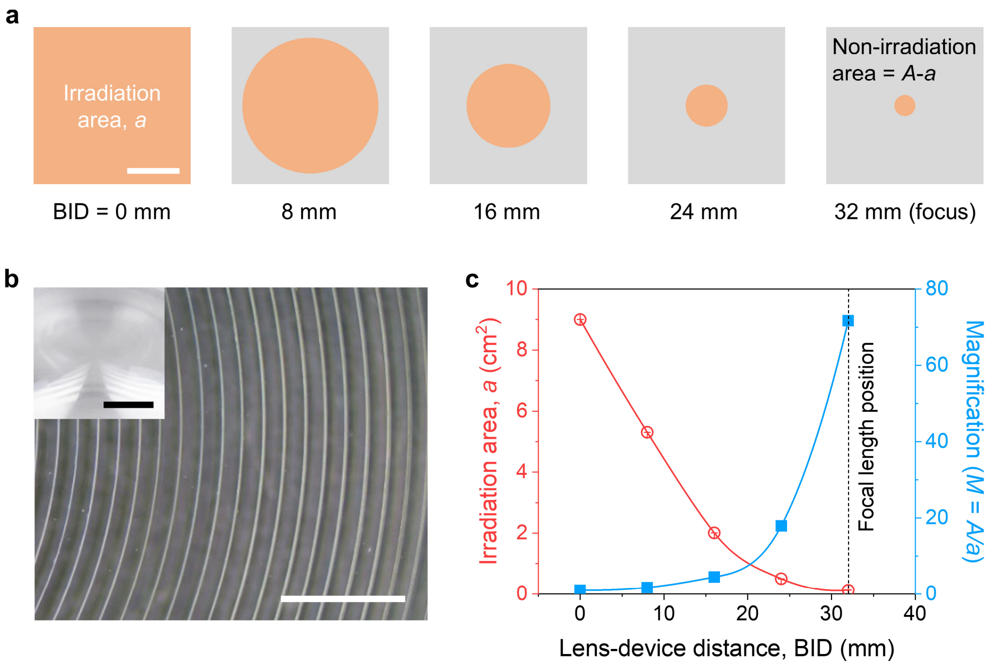

Supplementary Fig. 12 Light illumination area and optical profiles. (a) Schematic of measured irradiation area, *a*, versus back image distance (BID) or lens-device distance. Scale bar: 10 mm. **(b)** Morphological microstructures of PMMA lens (scale bar: 500 μm). Inset, photographic image, scale bar: 10 mm. **(c)** Irradiation area and magnification vary with the lens-device distance. The focal length of the Fresnel lens is 32 mm.

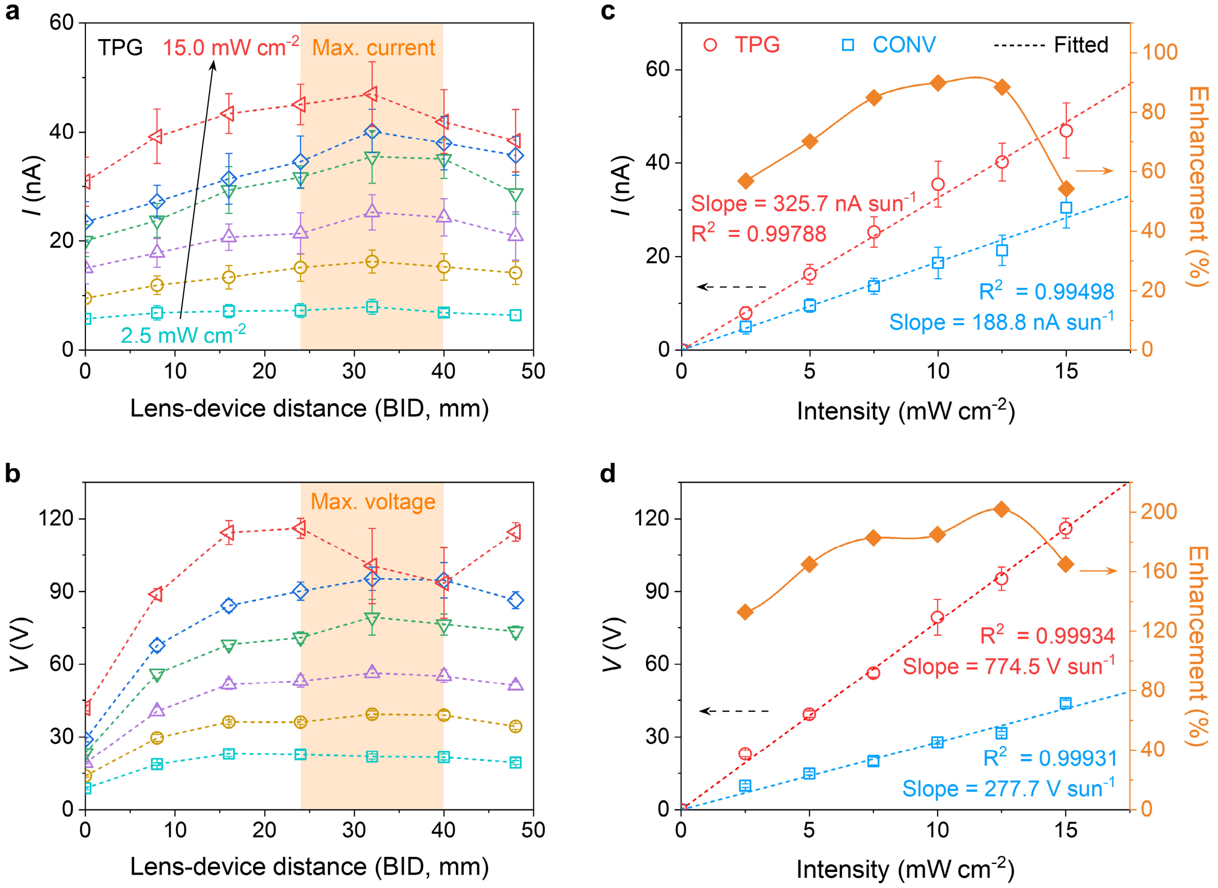

Supplementary Fig. 13 Full-parameter optimization of the electrical output of TPG. (a) Current and voltage **(b)** versus the lens-device distance under different intensities of 2.5-15 mW cm^{-2}, with an interval of 2.5 mW cm^{-2}. **(c)** The relationship between current and intensity for conventional and TPG measurement. The current enhancement for TPG was plotted on the right axis. **(d)** The relationship between voltage and intensity of conventional and TPG conditions. The voltage enhancement for TPG was plotted on the right axis.

a

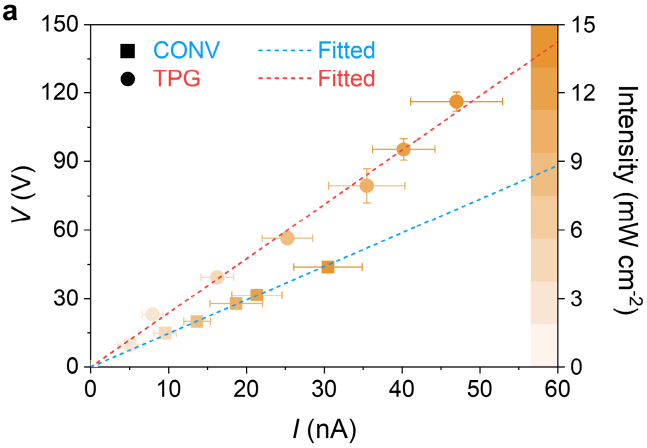

b

Equation	V=I*R
Plot	CONV
Resistance (GΩ)	1.47033 ± 0.02501
R-Square (COD)	0.99827

Equation	V=I*R
Plot	TPG
Resistance (GΩ)	2.3737 ± 0.04538
R-Square (COD)	0.99781

Supplementary Fig. 14 Current-voltage characteristics of pyroelectric output. (a) Voltage versus current of single conventional and TPG. **(b)** Following Ohm's law, the internal resistance of conventional and TPG is extracted.

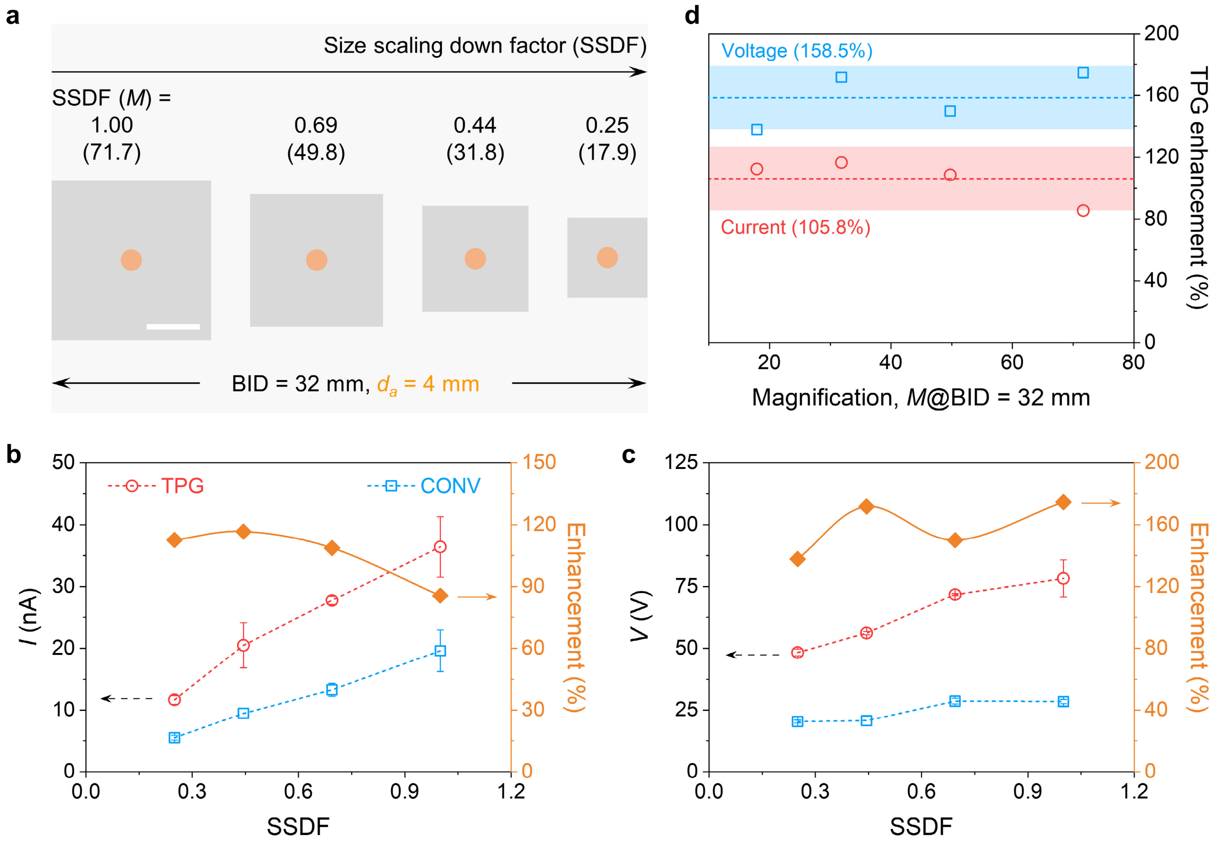

Supplementary Fig. 15 Enhanced output of size scaling down TPG. (a) Geometry diagram of TPG at various size scaling down factor (SSDF). The SSDF of TPG with a device area of 30 mm × 30 mm was defined as 1.0 for reference. Scale bar: 10 mm. **(b)** The relationship between current and SSDF for conventional and TPG measurement. The current enhancement for TPG was plotted on the right axis. **(c)** The relationship between voltage and SSDF of conventional and TPG conditions. The voltage enhancement for TPG was plotted on the right axis. **(d)** The relationship between TPG enhancement and magnifications at BID = 32 mm and d_a = 4 mm. The illumination is 10 mW cm^{-2}.

a

Inorganic pyroelectrics	$PMN_{0.7}PT_{0.3}$	PZT
Category	Relaxor ferroelectrics	Ferroelectric ceramics
Length (mm)	20	20
Width (mm)	20	20
Thickness (mm)	0.5	0.4
Electrodes	Au	Ag
Solar absorber	CNT	CNT

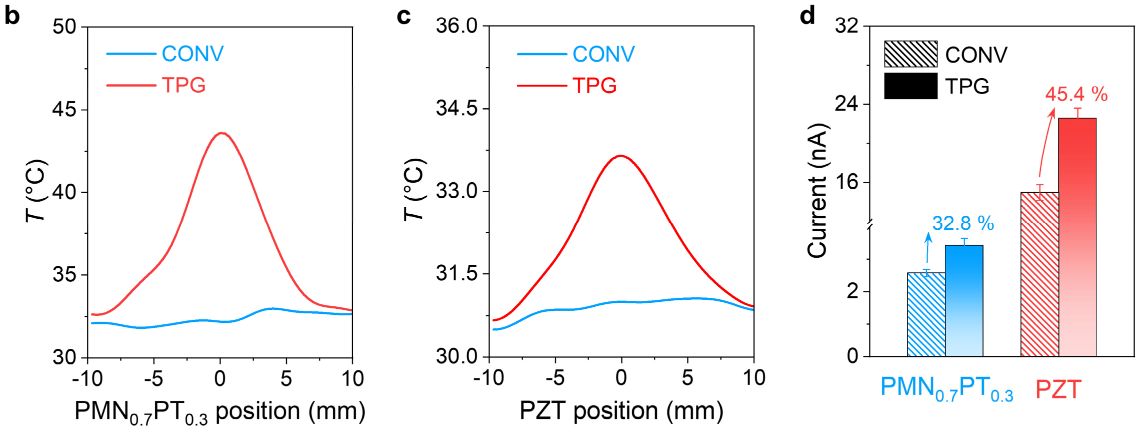

Supplementary Fig. 16 Enhanced output of TPG using inorganic ceramics. (a) Parameters of lead magnesium niobate-lead titanate relaxor ferroelectric ceramic ($PMN_{0.7}PT_{0.3}$) and lead zirconium titanate (PZT) ferroelectric ceramic devices. Temperature profiles for conventional and TPG measurements of **(b)** $PMN_{0.7}PT_{0.3}$, as well as **(c)** PZT devices. **(d)** Current enhancement of TPG in contrast to conventional devices. The illumination is 10 mW cm^{-2}.

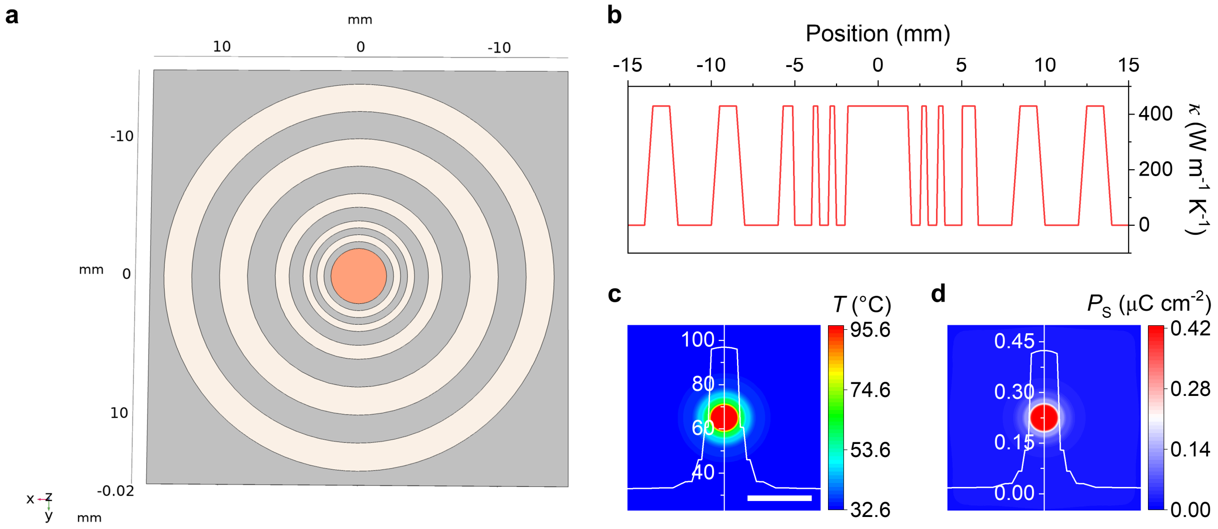

e

Pyroelectric input & output parameters	CONV	TPG	*κ*-TPG	Extracted methods
Input solar intensity, q_{solar}	10 mW cm^{-2}			/
Magnification, M	1	71.7		/
Thermal conductivity, κ	0.5		0.5, 429	Solar absorber
Input heat flux, q_{input}	9M mW cm^{-2} (81 mW)			Absorbance of 0.9
$P_{S@A}$ (μC cm^{-2})	0.3	1.1	3.0	2D Integrate (area A)
$P_{S@a}$ (μC cm^{-2})	0.3	0.8	1.1	2D Integrate (area a)
$P_{S@A}/P_{S@a}$	1.0	1.4	2.7	/

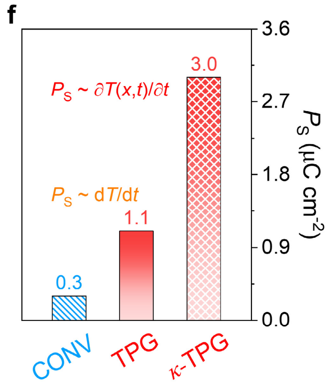

Supplementary Fig. 17 Enhanced output of TPG using asymmetrical solar absorbers with distinctive inhomogeneous thermal conductivities (*κ*-TPG). (a) Geometry of *κ*-TPG with alternate different solar absorber materials (CNT and Silver). **(b)** Corresponding thermal conductivity profile across the solar absorber. **(c)** Temperature and **(d)** polarization profiles. Scale bar: 1 cm. **(e)** Comparison of polarization increment for conventional, TPG, and *κ*-TPG devices. **(f)** Net *P*s of conventional, TPG, and *κ*-TPG devices.

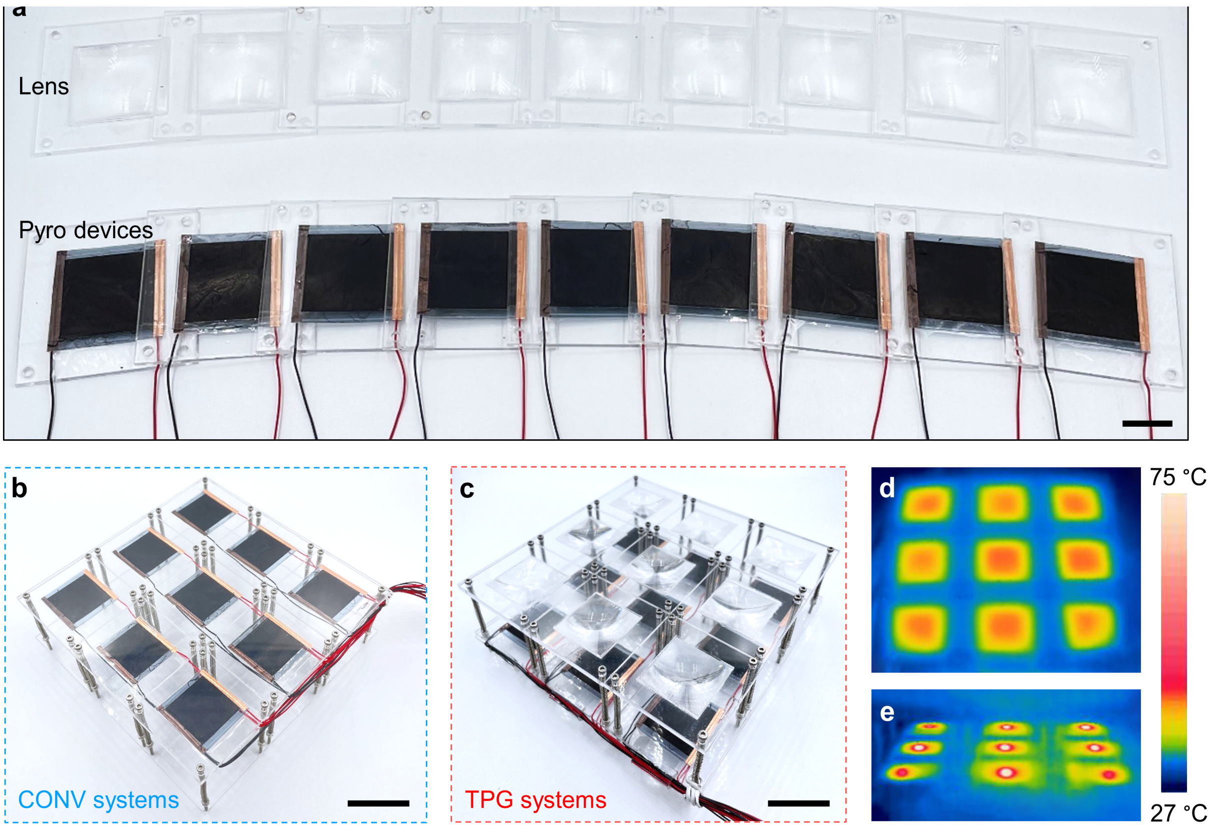

Supplementary Fig. 18 Conventional and TPG systems for the outdoor test. (a) TPG components. Top row, Fresnel lenses; bottom row, PVDF-based units. Scale bar: 1.5 cm. Modular and scalable conventional **(b)** and **(c)** TPG systems. Scale bar: 3.0 cm. Infrared images of **(d)** conventional and **(e)** TPG systems under 10 mW cm^{-2} illumination.

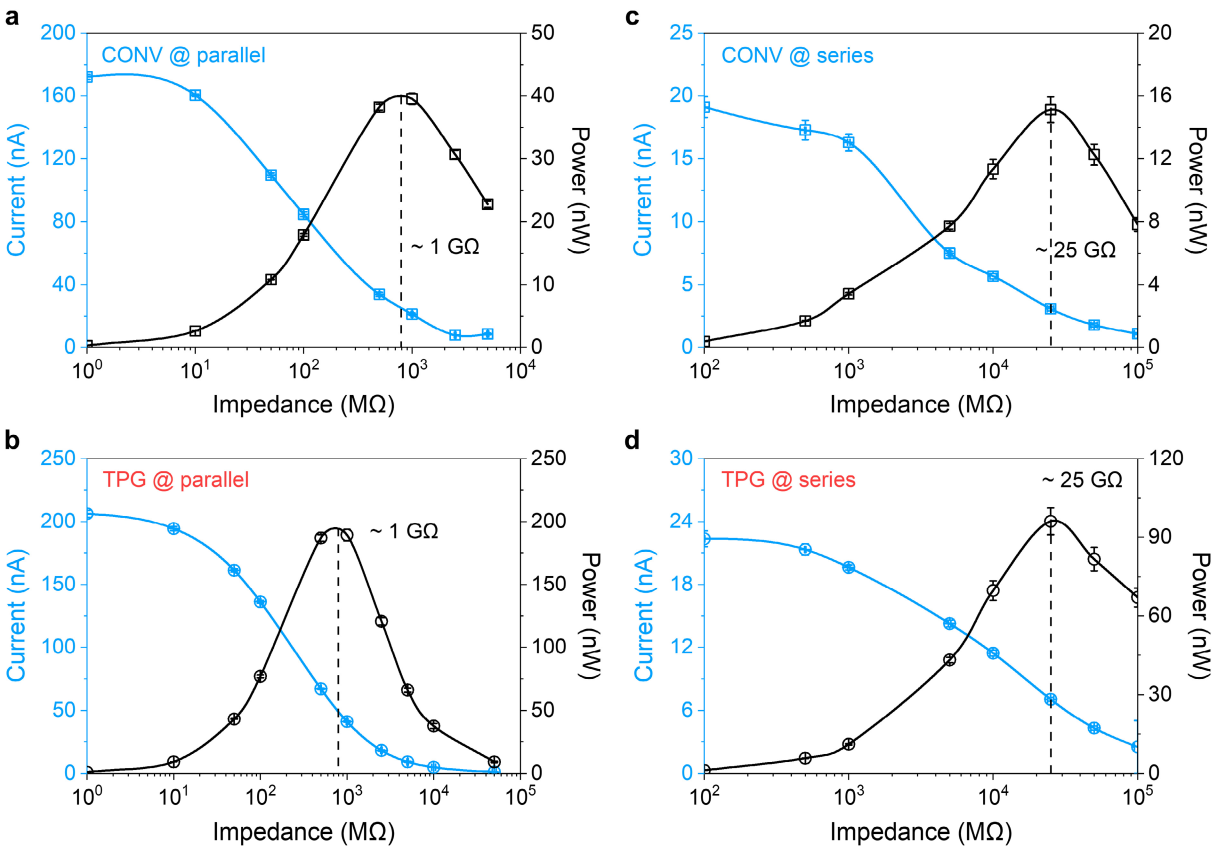

Supplementary Fig. 19 Load characteristics and output power of scalable conventional and TPG systems. Output current and power vary with different impedances in parallel for conventional **(a)** and **(b)** TPG systems. The matched impedance for the modular system in parallel is ~ 1 GΩ. The electrical output varies with different impedances in series for conventional **(c)** and **(d)** TPG systems. The matched impedance for the modular system in series is ~ 25 GΩ. The illumination is 10 mW cm^{-2}.

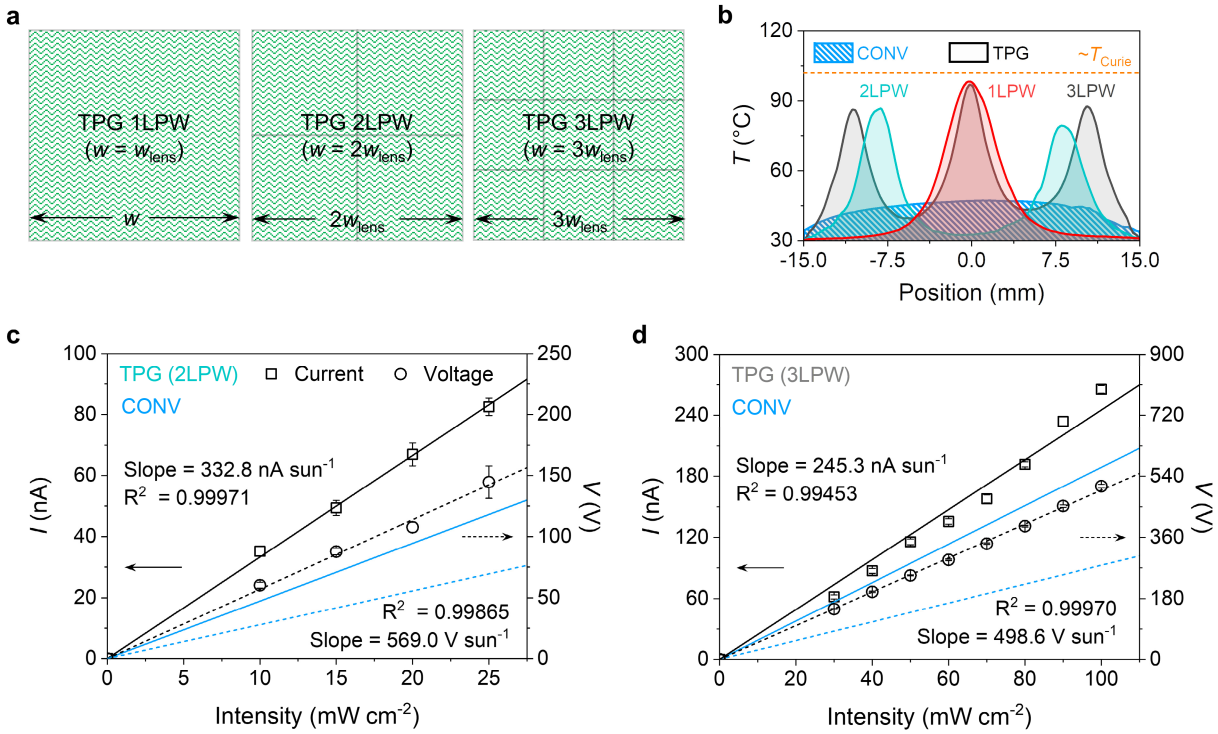

Supplementary Fig. 20 Enhanced TPG using multiple transverse heat ripples. (a) Geometry diagram of multiple lenses. LPW, lenses per width (device). **(b)** Temperature profiles of the conventional device (100 mW cm^{-2}) and TPG with 1LPW (10 mW cm^{-2}), 2LPW (25 mW cm^{-2}), 3LPW (100 mW cm^{-2}). Output current and voltage of TPG at **(c)** 2LPW and **(d)** 3LPW as compared with the conventional device under various illuminations.

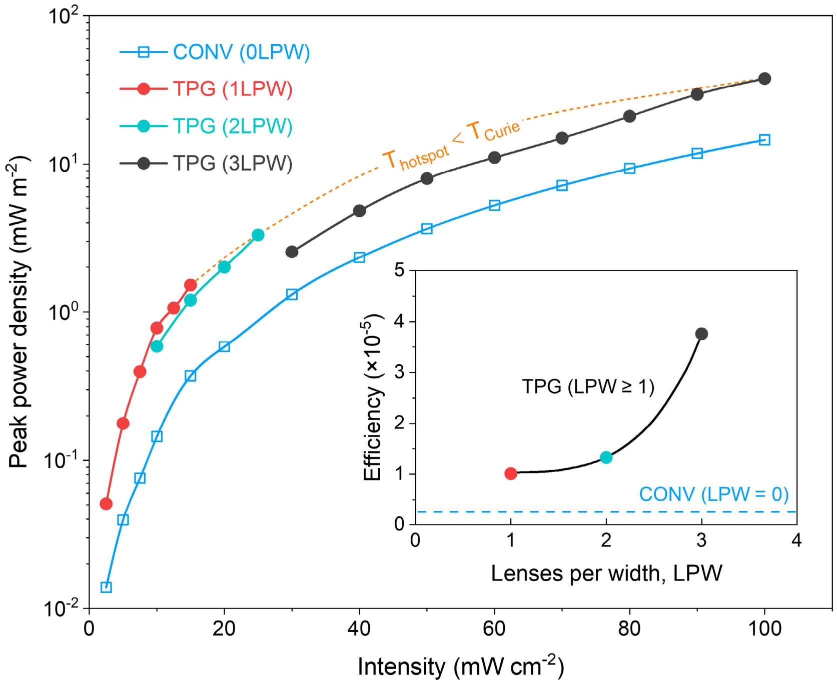

Supplementary Fig. 21 Peak power density of conventional and TPG devices versus illumination intensities. Inset, the energy conversion efficiency of TPG versus LPW. LPW, lenses per width (device).

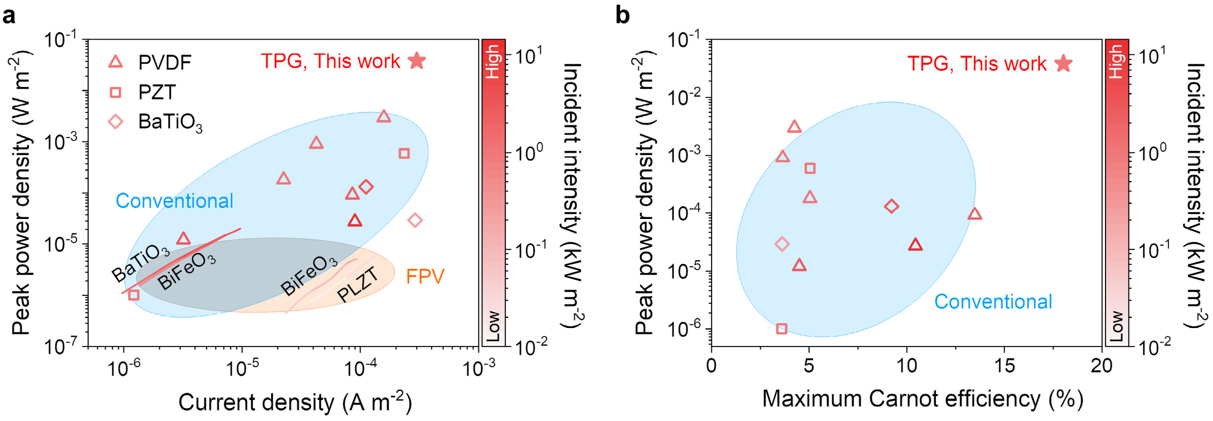

Supplementary Fig. 22 Comparison of the power output of ferrophotovoltaics, conventional pyroelectrics, and proposed TPG device. Peak power density versus (a) current density and (b) maximum Carnot efficiency. The color contrast of scatters and lines represents the illumination intensity. Reported data can be found in Supplementary Table 3.

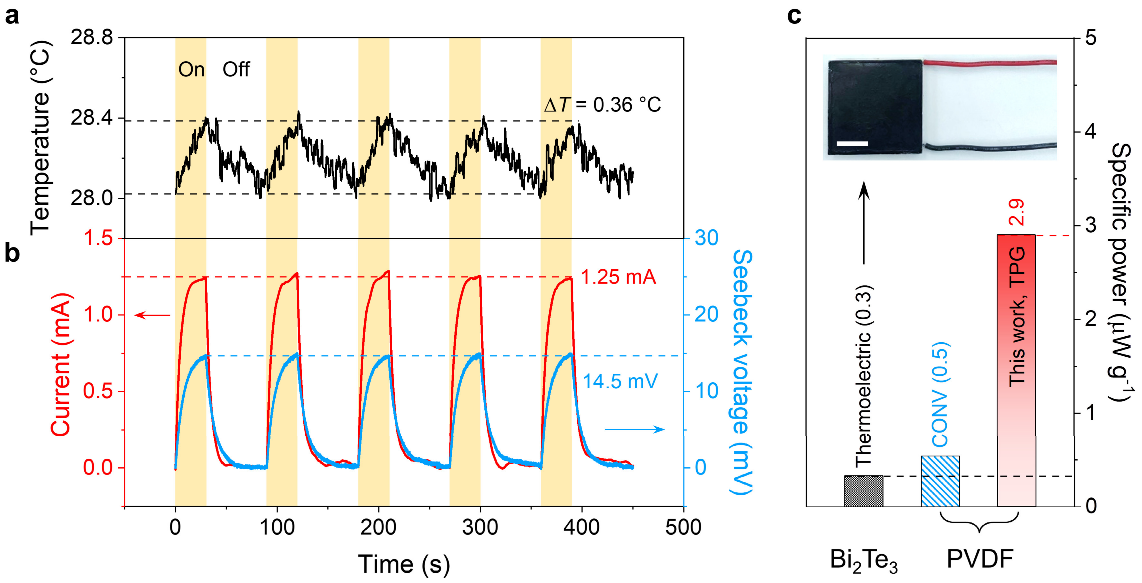

Supplementary Fig. 23 Measurement of solar thermoelectric generation under heating/cooling process. **(a)** The absorber temperature, **(b)** current and voltage output, and **(c)** comparison of specific power. Inset, commercial bismuth telluride device with a dimension of 30 mm × 30 mm × 4 mm. Scale bar: 1 cm. The specific power was calculated from the ratio of peak output power and device weight (Bi_2Te_3-based thermoelectric device, 13.740g; PVDF-based pyroelectric device, 0.241g). Measurement details were similar to that of pyroelectric devices. The illumination is 10 mW cm^{-2}. The modest temperature variation of thermoelectrics (Bi_2Te_3) in contrast to the larger variation of conventional pyroelectrics (PVDF, Supplementary Fig. 3a) under identical solar illumination conditions is mainly ascribed to distinctive specific heat capacities and mass weights. Under consistent solar irradiance (10 mW cm^{-2}), solar absorbance (CNT coating layer), and measured conditions (28 °C, RH 60%), a smaller heat capacity ($C_p m$), where C_p represents the specific heat capacity and m represents mass, contributes to achieving a larger ΔT. Thereby, the temperature variation of conventional PVDF-based pyroelectrics (m = 0.241g, $C_p \approx 1.2$ J g^{-1} K^{-1}) is larger than that of Bi_2Te_3-based thermoelectric device (m = 13.740g, $C_p \approx 0.2$ J g^{-1} K^{-1}).

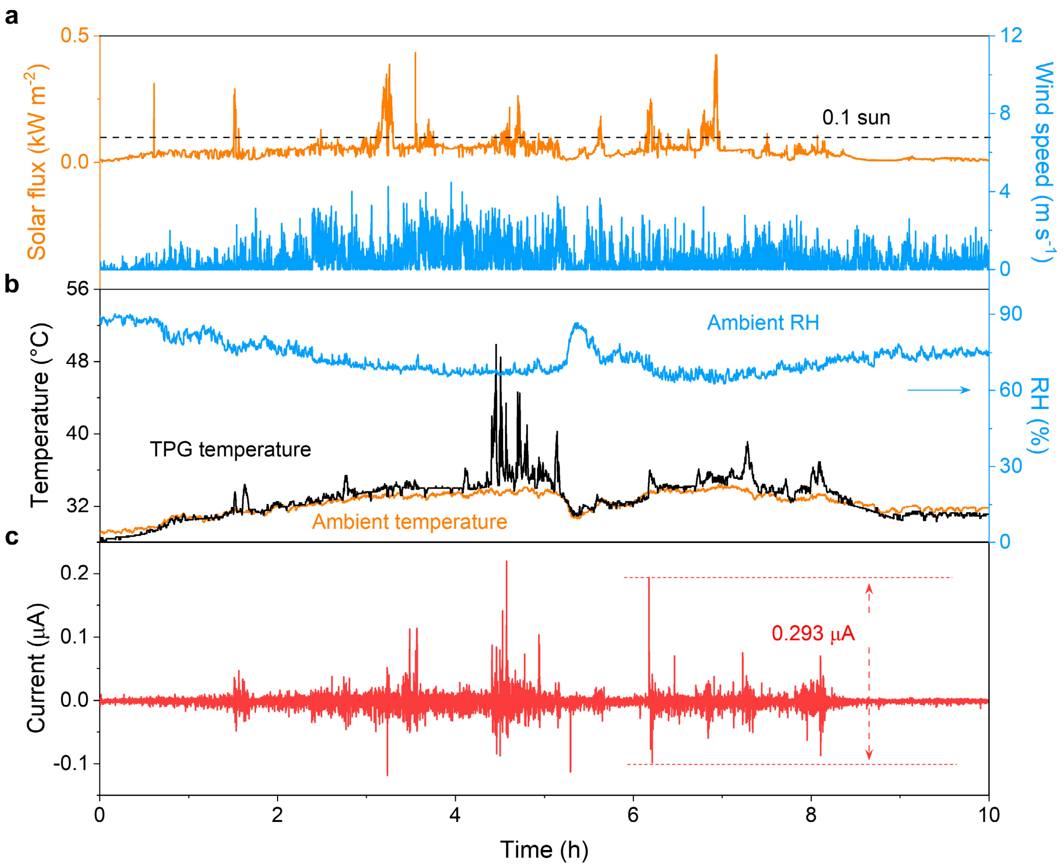

Supplementary Fig. 24 Outdoor-under-tree-shadow test of scalable TPG system. (a) Temporal variation of solar flux, wind speed, and **(b)** ambient temperature, TPG temperature (illuminated hotspot), ambient RH and **(c)** harvested current (in parallel).

Supplementary Table 1. Parameters for conventional and transverse pyroelectricity simulations

Parameters	Conventional	TPG	κ-TPG
PVDF dimension (mm)		30.0 × 30.0 × 0.08	
PVDF/CNT emissivity/ε		0.9	
Thermal penetration/diffusion depth (mm)		0.01†	
Magnification/M	1	71.7	
Illuminated hotspot diameter/d_a (mm)	/	4	
Ambient temperature/T_{amb} (°C)		28	
Ambient relative humidity/RH (%)		60	
Heat convection coefficient/h (W m^{-2} °C^{-1})		5-10	

† where the thermal penetration/diffusion depth of PVDF-based thin film is calculated from reference [30].

Supplementary Table 2. Calculation of gain factors for conventional and TPG devices

Parameters	Conventional		TPG		Gain factor	
	Heating	Cooling	Heating	Cooling	Conventional	TPG
$(\mathrm{d}T/\mathrm{d}t)_{\mathrm{abs}}$ (°C s^{-1})	0.546	0.522	13.910	18.031	0.955	1.296
I_{abs} (nA)	9.761	11.570	13.061	20.102	1.049	1.762
p (μC m^{-2} °C^{-1})	41.067	41.067	41.067	90.042	1	2.193
$q_{\mathrm{in-plane}}$ (W cm^{-2})	0.009	0.009	0.009	0.117	1	13.170
$q_{\mathrm{out-of-plane}}$ (W cm^{-2})	0.002	0.002	0.002	0.002	1	1

From a view of the pyroelectric thermodynamics (Fig. 1a, 1b, and Supplementary Fig. 2a), the boosted TPG output from the enhanced cooling process can be revealed from the dipole moment shift angle (θ) under temporal temperature fluctuations as well[31,32]. Typically, α, β, γ represent the single dipole moment shift angle at thermal equilibrium, heating, and cooling conditions, respectively, and the relationship $\beta > \alpha > \gamma$ is applicable for general pyroelectrics. Specifically, for conventional pyroelectrics under uniform heat variation, where $\beta - \alpha \approx \alpha - \gamma$ indicates the consistent heating/cooling processes[33,34]. By contrast, for proposed TPG, the in-plane quasi-static conductive heat flux and transverse heat ripple propagation produce a large dipole moment shift angle at the cooling condition, i.e., $\alpha - \gamma > \beta - \alpha$; this originally illustrates why the gain factor of TPG outperforms that of the conventional device.

Supplementary Table 3. State-of-the-art comparison of solar pyroelectrics, ferrophotovoltaics, and thermoelectrics

Device	Size a×b×c (mm^3)	Solar intensity (kW m^{-2})	T_h, T_c (°C)	Current density (A m^{-2})	Peak power density‡ (W m^{-2})	Power density (W cm^{-3})	η_{Carnot} (%)	Conventional/ TPG	Refs
PVDF	20×20×0.11	1.00	22.8, 34.0	42.5μ	0.9m	8.21μ	3.6	Conventional	[29]
PVDF	75×75×0.08	1.00	44.0, 28.0	22.5μ	0.18m	2.26μ	5.0	Conventional	[35]
PVDF	12×10×0.09	1.00	56.0, 42.0	0.16m	2.95m	31.3μ	4.2	Conventional	[36]
PVDF	50×50×0.27	2.26	75.0, 28.8	85.6μ	92.0μ	0.35μ	0.9	Conventional	[37]
PVDF	40×40×0.11	3.06	42.2, 28.0	3.2μ	12.0μ	1.09μ	4.5	Conventional	[38]
PVDF	20×5×0.10	14.5	18.0, 52.0	90.0μ	27.0μ	2.7μ	10.4	Conventional	[39]
PZT	*Φ*35×0.37†	1.00	48.0, 60.0	1.2μ	1.0μ	2.7n	3.6	Conventional	[40]
PZT	*Φ*24×0.14†	1.00	27.5, 43.5	0.24m	0.59m	4.2μ	5.0	Conventional	[41]
BTO	*Φ*20×0.85†	0.25	27.9, 39.2	0.29m	29.0μ	34.1n	3.6	Conventional	[42]
BTO	*Φ*10×0.85†	2.47	51.9, 21.8	0.11m	0.13m	0.15μ	3.6	Conventional	[43]
PVDF	30×30×0.08	0.15	29.6, 33.0	32.9μ	0.37m	4.64μ	1.2	Conventional	***This work***
PVDF	30×30×0.08	0.15	29.8, 118.8	52.2μ	1.52m	19.0μ	22.8	TPG	***This work***
PVDF	30×30×0.08	1.00	30.3, 97.0	0.30m	37.6m	470.5μ	18.0	TPG	***This work***

† Where *ϕ* stands for the diameter of circular devices.

‡The peak power density of ferrophotovoltaics (FPV) ($BiFeO_3$[8,44], PLZT[45], $BaTiO_3$[46]) was estimated using equation (S4). The peak power density of solar organic thermoelectric generators (SOTEG) (PEDOT:PSS[47], Cu_2S/phenol[48], and PEDOT:PAMPS[49]) was extracted using quadratic correlation[50].

Supplementary Movie 1. Outdoor test of transverse pyroelectric generation systems

Supplemental References

1. Bowen, C. R. *et al.* Pyroelectric materials and devices for energy harvesting applications. *Energ. Environ. Sci.* **7**, 3836-3856, (2014).
2. Kim, J. *et al.* High-Performance Piezoelectric, Pyroelectric, and Triboelectric Nanogenerators Based on P(VDF-TrFE) with Controlled Crystallinity and Dipole Alignment. *Adv. Funct. Mater.* **27**, 1700702, (2017).
3. Jachalke, S. *et al.* How to measure the pyroelectric coefficient? *Appl. Phys. Rev.* **4**, 021303, (2017).
4. Jiang, J. *et al.* Giant pyroelectricity in nanomembranes. *Nature* **607**, 480-485, (2022).
5. Das-Gupta, D. K. On the nature of pyroelectricity in polyvinylidene fluoride. *Ferroelectrics* **33**, 75-89, (1981).
6. Karthik, J., Agar, J. C., Damodaran, A. R. & Martin, L. W. Effect of 90 degrees domain walls and thermal expansion mismatch on the pyroelectric properties of epitaxial PbZr0.2Ti0.8O3 thin films. *Phys. Rev. Lett.* **109**, 257602, (2012).
7. Moalla, R. *et al.* Large anisotropy of ferroelectric and pyroelectric properties in heteroepitaxial oxide layers. *Sci. Rep.* **8**, 4332, (2018).
8. Yang, S. Y. *et al.* Above-bandgap voltages from ferroelectric photovoltaic devices. *Nat. Nanotechnol.* **5**, 143-147, (2010).
9. Raeder, T. M. *et al.* Anisotropic in-plane dielectric and ferroelectric properties of tensile-strained BaTiO3 films with three different crystallographic orientations. *AIP Adv.* **11**, 025016, (2021).
10. Aruchamy, N. *et al.* Influence of substrate stress on in-plane and out-of-plane ferroelectric properties of PZT films. *J. Appl. Phys.* **131**, 014101, (2022).
11. Matsuo, H. & Noguchi, Y. High Photocurrent Anisotropy in Domain-Engineered Ferroelectrics for Visible-Light Polarization Detection. *Adv. Opt. Mater.* **10**, 2201280, (2022).

12. Aleksandrova, M., Sohan, A., Kollu, P. & Dobrikov, G. Pyroelectric Properties of Ba(x)Sr((1-x))TiO(3)/PVDF-TrFE Coating on Silicon. *Membranes (Basel)* **11**, 577, (2021).
13. Gavrilova, N. D., Drozhdin, S. N., Novik, V. K. & Maksimov, E. G. Relationship between the pyroelectric coefficient and the lattice dynamics of the pyroelectrics. *Solid State Commun.* **48**, 129-133, (1983).
14. Szigeti, B. Temperature Dependence of Pyroelectricity. *Phys. Rev. Lett.* **35**, 1532-1534, (1975).
15. Xu, X. *et al.* Femtosecond laser writing of lithium niobate ferroelectric nanodomains. *Nature* **609**, 496-501, (2022).
16. Dishon Ben Ami, S. *et al.* Engineering of Pyroelectric Crystals Decoupled from Piezoelectricity as Illustrated by Doped alpha-Glycine. *Angew. Chem. Int. Ed. Engl.* **61**, e202213955, (2022).
17. Poosanaas, P., Dogan, A., Thakoor, S. & Uchino, K. Influence of sample thickness on the performance of photostrictive ceramics. *J. Appl. Phys.* **84**, 1508-1512, (1998).
18. Scientific ReportsLi, W. *et al.* Improper molecular ferroelectrics with simultaneous ultrahigh pyroelectricity and figures of merit. *Sci. Adv.* **7**, eabe3068, (2021).
19. Pandya, S. *et al.* Pyroelectric energy conversion with large energy and power density in relaxor ferroelectric thin films. *Nat. Mater.* **17**, 432-438, (2018).
20. Lheritier, P. *et al.* Large harvested energy with non-linear pyroelectric modules. *Nature* **609**, 718-721, (2022).
21. Vincent Ming Hong, N. *et al.* in *Comprehensive Energy Systems* Vol. 2 *Pyroelectric Materials* (ed Ibrahim Dincer) Ch. 2.23, 720-759 (Elsevier, 2018).
22. Damjanovic, D. Ferroelectric, dielectric and piezoelectric properties of ferroelectric thin films and ceramics. *Rep. Prog. Phys.* **61**, 1267-1324, (1998).
23. Sasabe, H., Nakayama, T., Kumazawa, K., Miyata, S. & Fukada, E. Photovoltaic Effect in Poly(vinylidene fluoride). *Polym. J.* **13**, 967-973, (1981).

24. Zhou, Y. *et al.* Dynamic piezo-thermoelectric generator for simultaneously harvesting mechanical and thermal energies. *Nano Energy* **69**, 104397, (2020).
25. Yang, M. M. *et al.* Piezoelectric and pyroelectric effects induced by interface polar symmetry. *Nature* **584**, 377-381, (2020).
26. Bowen, C. R., Kim, H. A., Weaver, P. M. & Dunn, S. Piezoelectric and ferroelectric materials and structures for energy harvesting applications. *Energ. Environ. Sci.* **7**, 25-44, (2014).
27. Yang, J. *et al.* Epitaxy Enhancement of Piezoelectric Properties in P(VDF-TrFE) Copolymer Films and Applications in Sensing and Energy Harvesting. *Adv. Electron. Mater.* **6**, 2000578, (2020).
28. Zhang, K., Wang, Y., Wang, Z. L. & Yang, Y. Standard and figure-of-merit for quantifying the performance of pyroelectric nanogenerators. *Nano Energy* **55**, 534-540, (2019).
29. Ding, T. *et al.* Hybrid Photothermal Pyroelectric and Thermogalvanic Generator for Multisituation Low Grade Heat Harvesting. *Adv. Energ. Mater.* **8**, 1802397, (2018).
30. Boccaccio, T., Bottino, A., Capannelli, G. & Piaggio, P. Characterization of PVDF membranes by vibrational spectroscopy. *J. Membr. Sci.* **210**, 315-329, (2002).
31. Born, M. On the Quantum Theory of Pyroelectricity. *Rev. Mod. Phys.* **17**, 245-251, (1945).
32. Lang, S. B. & Das-Gupta, D. K. in *Handbook of Advanced Electronic and Photonic Materials and Devices* Vol. 4 *Pyroelectricity: Fundamentals and applications* (ed Hari Singh Nalwa) Ch. 1, 1-55 (Academic Press, 2001).
33. Li, H., Bowen, C. R. & Yang, Y. Scavenging Energy Sources Using Ferroelectric Materials. *Adv. Funct. Mater.* **31**, 2100905, (2021).
34. Kishore, R. A. in *Ferroelectric Materials for Energy Harvesting and Storage* Vol. Electronic and Optical Materials (eds Deepam Maurya, Abhijit Pramanick, & Dwight Viehland) Ch. 3, 85-106 (Woodhead Publishing, 2021).
35. Wang, X. Q. *et al.* Nanophotonic-Engineered Photothermal Harnessing for Waste Heat

Management and Pyroelectric Generation. *ACS Nano* **11**, 10568-10574, (2017).

36. Lee, J. *et al.* Enhanced pyroelectric conversion of thermal radiation energy: Energy harvesting and non-contact proximity sensor. *Nano Energy* **97**, 107178, (2022).
37. Gokana, M. R., Wu, C.-M., Motora, K. G., Qi, J. Y. & Yen, W.-T. Effects of patterned electrode on near infrared light-triggered cesium tungsten bronze/poly(vinylidene)fluoride nanocomposite-based pyroelectric nanogenerator for energy harvesting. *J. Power Sources* **536**, 231524, (2022).
38. Park, T. *et al.* Photothermally Activated Pyroelectric Polymer Films for Harvesting of Solar Heat with a Hybrid Energy Cell Structure. *ACS Nano* **9**, 11830-11839, (2015).
39. Zhao, T. *et al.* An infrared-driven flexible pyroelectric generator for non-contact energy harvester. *Nanoscale* **8**, 8111-8117, (2016).
40. Azad, P., Khushboo & Vaish, R. Solar Energy Harvesting Using Pyroelectric Effect Associated with Piezoelectric Buzzer. *Phys. Status Solidi A* **216**, 1900440, (2019).
41. Zhang, Q., Agbossou, A., Feng, Z. & Cosnier, M. Solar micro-energy harvesting with pyroelectric effect and wind flow. *Sensor Actuat. A-Phys.* **168**, 335-342, (2011).
42. Ma, N., Zhang, K. & Yang, Y. Photovoltaic-Pyroelectric Coupled Effect Induced Electricity for Self-Powered Photodetector System. *Adv. Mater.* **29**, 1703694, (2017).
43. Song, K., Ma, N., Mishra, Y. K., Adelung, R. & Yang, Y. Achieving Light-Induced Ultrahigh Pyroelectric Charge Density Toward Self-Powered UV Light Detection. *Adv. Electron. Mater.* **5**, 1800413, (2019).
44. Guo, R. *et al.* Non-volatile memory based on the ferroelectric photovoltaic effect. *Nat. Commun.* **4**, 1990, (2013).
45. Poosanaas, P., Tonooka, K. & Uchino, K. Photostrictive actuators. *Mechatronics* **10**, 467-487, (2000).
46. Spanier, J. E. *et al.* Power conversion efficiency exceeding the Shockley–Queisser limit in a ferroelectric insulator. *Nat. Photonics* **10**, 611-616, (2016).

47. Jurado, J. P. *et al.* Solar Harvesting: a Unique Opportunity for Organic Thermoelectrics? *Adv. Energ. Mater.* **9**, 1902385, (2019).
48. Li, K. *et al.* Enhanced thermoelectric performance and tunable polarity in 2D Cu2S-phenol superlattices composites for solar energy conversion. *Nano Energy* **84**, 105902, (2021).
49. Cho, C., Kim, B., Park, S. & Kim, E. Bisulfate transport in hydrogels for self-healable and transparent thermoelectric harvesting films. *Energ. Environ. Sci.* **15**, 2049-2060, (2022).
50. Zhou, Y., Guo, Z. & He, J. Redesign high-performance flexible thermoelectrics: From mathematical algorithm to artificial cracks. *Appl. Phys. Lett.* **116**, 043904, (2020).
