# Supplementary material for: Giant polarization ripple in transverse pyroelectricity
Source: Nat Commun. 2023 Jan 26;14:426. doi: 10.1038/s41467-023-35900-x (PMC9879950; doi:10.1038/s41467-023-35900-x)
Supplement: Supplementary file 2 — Description of Additional Supplementary Files [file 41467_2023_35900_MOESM2_ESM.pdf]

Description of Additional Supplementary Files

Supplementary Movie 1. Outdoor test of transverse pyroelectric generation systems
